# Dual inhibition of protein kinase C and p53-MDM2 or PKC and mTORC1 are novel efficient therapeutic approaches for uveal melanoma

**DOI:** 10.18632/oncotarget.9552

**Published:** 2016-05-22

**Authors:** Guillaume Carita, Estelle Frisch-Dit-Leitz, Ahmed Dahmani, Chloé Raymondie, Nathalie Cassoux, Sophie Piperno-Neumann, Fariba Némati, Cécile Laurent, Leanne De Koning, Ensar Halilovic, Sebastien Jeay, Andrew Wylie, Caroline Emery, Sergio Roman-Roman, Marie Schoumacher, Didier Decaudin

**Affiliations:** ^1^ Laboratory of preclinical investigation, Department of Translational Research, PSL University, Institut Curie, Paris, France; ^2^ Department of Translational Research, Institut Curie, PSL University, Paris, France; ^3^ Department of Ophthalmological Oncology, Institut Curie, Paris, France; ^4^ Department of Medical Oncology, Institut Curie, Paris, France; ^5^ Residual Tumor & Response to Treatment Lab, Department of Translational Research, Institut Curie, PSL University, Paris, Paris, France; ^6^ RPPA Platform, Department of Translational Research, Institut Curie, PSL University, Paris, France; ^7^ Novartis Institutes for Biomedical Research, Cambridge, MA USA

**Keywords:** xenograft models, cellular response to anticancer drugs, uveal melanoma, AEB071 combinations, synergy

## Abstract

Uveal melanoma (UM) is the most common cancer of the eye in adults. Many UM patients develop metastases for which no curative treatment has been identified. Novel therapeutic approaches are therefore urgently needed. UM is characterized by mutations in the genes *GNAQ and GNA11* which activate the PKC pathway, leading to the use of PKC inhibitors as a rational strategy to treat UM tumors. Encouraging clinical activity has been noted in UM patients treated with PKC inhibitors. However, it is likely that curative treatment regimens will require a combination of targeted therapeutic agents. Employing a large panel of UM patient-derived xenograft models (PDXs), several PKC inhibitor-based combinations were tested *in vivo* using the PKC inhibitor AEB071. The most promising approaches were further investigated *in vitro* using our unique panel of UM cell lines. When combined with AEB071, the two agents CGM097 (p53-MDM2 inhibitor) and RAD001 (mTORC1 inhibitor) demonstrated greater activity than single agents, with tumor regression observed in several UM PDXs. Follow-up studies in UM cell lines on these two drug associations confirmed their combination activity and ability to induce cell death. While no effective treatment currently exists for metastatic uveal melanoma, we have discovered using our unique panel of preclinical models that combinations between PKC/mTOR inhibitors and PKC/p53-MDM2 inhibitors are two novel and very effective therapeutic approaches for this disease. Together, our study reveals that combining PKC and p53-MDM2 or mTORC1 inhibitors may provide significant clinical benefit for UM patients.

## INTRODUCTION

With an incidence of about 2-8 cases per million per year in western countries, uveal melanoma (UM) remains a rare malignancy but constitutes the most common primary intraocular tumor in adults [1]. UM arises in the pigmented uveal tract including the choroid, ciliary body and iris [2]. Despite a 10-year local control rate of 95%, metastases occur in about one third of patients, developing mainly in the liver (89% of cases) [3]. As a consequence, the median survival time of metastatic UM patients ranges from 3 to 12 months [4][5]. Metastatic risk has been associated with monosomy of chromosome 3 and loss of expression of the protein BAP1 [6][7]. Systemic therapy with alkylating agents have shown only modest efficacy [8] and no systemic treatment has been capable of increasing survival. New therapeutic approaches and particularly new targeted therapies are therefore warranted [2][5][9].

More than 80% of UM have mutations in the genes *GNAQ* and *GNA11*, which encode for small GTPases [10]. These mutations are mutually exclusive, affecting 46% and 35% of UM cases respectively [11][12][13]. Oncogenic signaling as a result of GNAQ/11 mutations is reported to hyperactivate the PLCβ/PKC/MAPK pathway (14][15][16]. Indeed, an anti-proliferative effect has been observed *in vitro* using both PKC and MEK inhibitors [16][17]. While the PKCi AEB071 could induce a *GNAQ^Q209L^*-dependent tumor growth inhibition *in vivo*, no sustained MAPK pathway inhibition could be achieved and inhibition of PKC alone was unable to trigger cell death *in vitro* and/or tumor regression *in vivo* [16]. Combination of AEB071 with the MEK inhibitor Binimetinib (MEK162) led to sustained inhibition of MAPK activity and significant *in vivo* tumor growth inhibition [16]. A phase I dose-escalation study of AEB071 in UM metastatic patients showed encouraging signs of clinical activity but overall the efficacy was relatively modest [18]. Two different MEK inhibitors have been investigated in clinical trials and showed a slight benefit for UM patients [19][20][21].

Our current knowledge of UM biology has led us to consider novel combination approaches, such as co-targeting PKC and the PI3K/AKT/mTOR pathway, MDM2/p53 signaling or cell cycle regulation. First, activation of the PI3K/AKT pathway in UM has been suggested by several reports [22][23][24] and anti-tumor activity has been observed in UM models using various PI3K/AKT/mTOR pathway inhibitors [25][26][27]. Moreover, a synergistic effect has been described after combination of AEB071 with the PI3Kα inhibitor BYL719 *in vitro* and *in vivo* [27]. Second, while *p53* mutations are not common in UM [28], several studies have shown that UM have an inactivated p53 pathway, due to (i) high expression of the protein MDM2 [28][29][30][31][32] and (ii) downregulation of the protein PERP in aggressive UM [33][34]. Furthermore, the MDM2 inhibitor Nutlin-3 was shown to reduce UM cell proliferation in a p53-dependent manner [35]. Third, a high cyclin D1 expression as well as a strong nuclear staining for Rb have been observed in UM patients [29][30][31], suggesting that targeting CDK4/6 activity could be a valuable therapeutic strategy.

Using a large panel of UM models [26][36][37], we evaluated combinations of the PKCi AEB071 with compounds targeting MEK1/2 (MEK162), p53-MDM2 (CGM097), mTORC1 (Everolimus/RAD001) and CDK4/6 (Ribociclib/LEE011). We first performed an *in vivo* combination screen in five different Patient-Derived Xenograft models (PDXs). Promising combinations were further investigated *in vitro* in our panel of UM cell lines with the goal to define the modality of action of these combinations and to build strong preclinical data for effective translation into UM clinical trials.

## RESULTS

### PKC and p53-MDM2 targeted inhibitors are consistently active in UM PDXs when dosed as single agents

We first evaluated the anti-tumor efficacy of AEB071 in five UM PDXs: MP42, MP46, MP55, MM33 and MM52 (Supplementary Figure S1A; Tables S1 and S2). AEB071 was orally administered twice daily at a dose of 120 or 240 mg/kg/day. A dose-dependent efficacy of AEB071 was observed in all models, with a significantly higher tumor growth inhibition (TGI) at the highest dose in all PDXs. The degree of AEB071 efficacy was variable depending on the PDXs with MP42 and MP46 models showing the highest sensitivity to PKCi.

With a view to evaluating AEB071-based combination regimens, four targeted agents were first tested as single agents in the same models. Compounds targeting MEK1/2 (MEK162), mTORC1 (RAD001), p53-MDM2 (CGM097) and CDK4/6 (LEE011) were tested alongside the lower AEB071 daily dose of 120 mg/kg to avoid any risk of toxicity when tested in combination. MEK162, RAD001 and CGM097 were tested in five PDXs while LEE011 was evaluated only in three models.

As shown in Supplementary Figure S1B and Table S3, treatment with MEK162 or LEE011 showed a modest TGI in the five PDX models from 13-50% for MEK162 or around 35% for LEE011. Treatment with RAD001 gave similar responses in three out of five PDXs but had a higher anti-tumor activity in MM33 and MM52, reaching a TGI of 70% and 71% respectively. Interestingly, treatment with CGM097 reduced tumor growth to a higher extent in all PDXs, from 56 to 90% of TGI. Notably, response to AEB071 treatment was similar to the previous dose-response experiment, except for one model (MP46). When looking at the overall response rate (ORR; see Supplementary Materials), AEB071, MEK162, LEE011, RAD001, CGM097 induced an ORR lower than −0.5 in 32%, 22%, 13%, 34%, and 70% respectively, confirming CGM097 as the most efficient agent (Supplementary Figure S2 and Table S3).

We next compared the overall efficacy across all tested compounds except LEE011. For each PDX model, we ranked the efficacy of all tested agents using a scoring method: a therapy was classified from 1 (more efficient treatment) to 4 (less efficient treatment) using the TGI criteria. The sum of the scores for each of the five PDXs was calculated for each compound and defined as the final score (Supplementary Table S4). While AEB071, MEK162 and RAD001 scored as 15, 14 and 13 respectively, the total score for CGM097 was 8, underlining the superior efficacy of this p53-MDM2 inhibitor over the other compounds, including AEB071.

### Dual inhibition of PKC with mTORC1 or PKC with p53-MDM2 induces significant anti-tumor effect in UM PDXs

**Figure 1:**
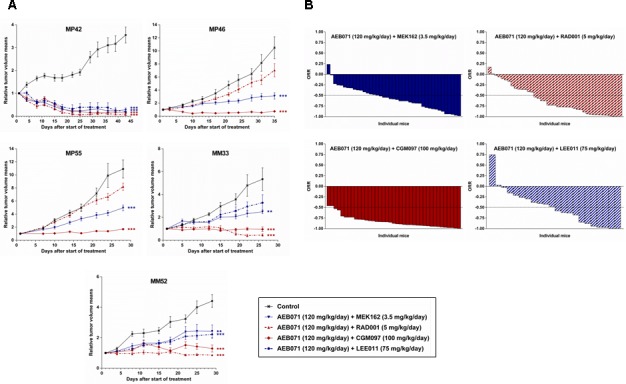
*In vivo* efficacy of AEB071-based combinations in UM PDXs **A.** Tumor growth curves, as mean of the RTV (relative tumor volume) ± SD. Doses for each compound are indicated in the legends. **: *p* < 0.01; ***: *p* < 0.001. **B.** Overall Response Rate (ORR) graphs.

**Figure 2:**
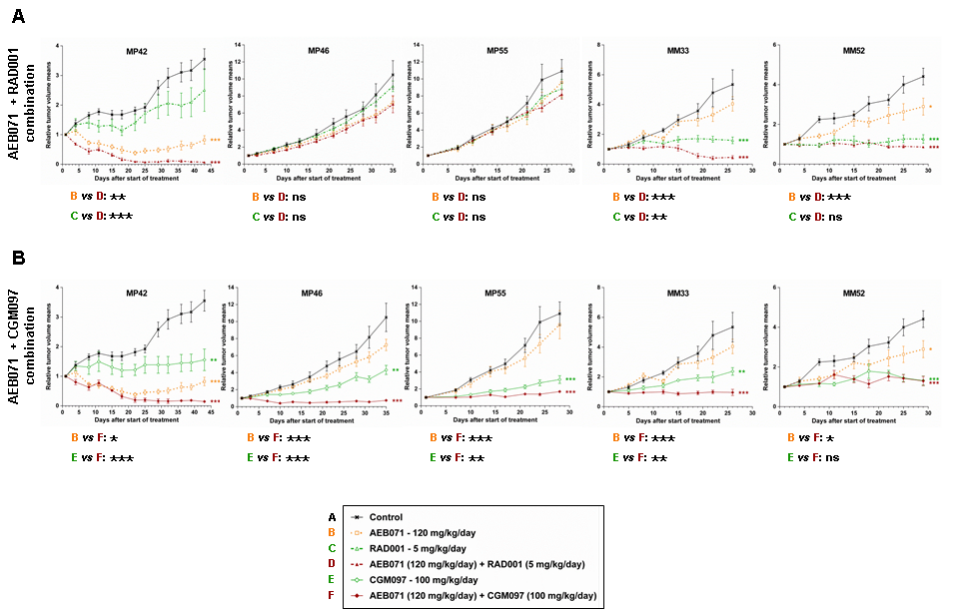
*In vivo* efficacy of AEB071 + RAD001 and AEB071 + CGM097 combinations in UM PDXs **A.** AEB071 + RAD001. **B.** AEB071 + CGM097. Tumor growth was evaluated by plotting the mean of the RTV (relative tumor volume) ± SD per group. Doses for each compound are indicated in the legends. *: *p* < 0.05; **: *p* < 0.01; ***: *p* < 0.001. ns: non-significant.

Results of the combinations between AEB071 and MEK162, RAD001, CGM097 or LEE011 are summarized in Figures 1 and 2, Supplementary Figures S3-S5 and Tables S5 and S6.

When comparing the efficacy of all combinations across the five PDX models, two combinations showed higher anti-tumor responses: AEB071 + RAD001 and AEB071 + CGM097 (Figures 1 and 2; Supplementary Table S5). Indeed, AEB071 + RAD001 co-treatment induced a significant TGI in three models with two tumor regressions (MP42, MM33) and one tumor stabilization (MM52). Combination activity was detected in two models (MP42 and MM33) (Figure 2). Strikingly, the AEB071 + CGM097 combination strongly reduced tumor growth, leading to tumor regression or stasis in all five PDX models. Combination activity was observed in four models (MP42, MP46, MP55, MM33) (Figure 2).

Similarly as AEB071 + CGM097, the AEB071 + MEK162 combination induced significant TGI in all five PDXs associated with combination activity in four models (Figure 1 and Supplementary Figure S4). However, its global anti-tumor effect was more modest than AEB071 + CGM097 treatment, with only one model showing tumor regression and a reduced tumor progression in the other PDXs. Treatment with AEB071 + LEE011 did not significantly enhance the effect of either AEB071 or LEE011 used alone (Figure 1 and Supplementary Figure S5).

We used our scoring system to compare the efficacy of all combinations except the one with LEE011. Combinations of AEB071 + MEK162, AEB071 + RAD001 and AEB071 + CGM097 scored as 13, 9 and 8 respectively (Supplementary Table S4). While assessment of monotherapy efficacy underlined the clear superiority of CGM097, two combinations appeared to be equivalent in our panel of UM PDXs, namely AEB071 + RAD001 and AEB071 + CGM097. The significant increase of efficacy of AEB071 + RAD001 combination compared to monotherapies suggests a possible synergistic effect between these two compounds, while co-treatment of AEB071 with CGM097 seems to be additive.

Overall, our *in vivo* findings show that targeting of PKC and p53-MDM2 or PKC and mTORC1 are effective combination strategies for *GNAQ/11* mutated UM PDXs, with tumor regressions often observed after PKC and p53-MDM2 inhibition.

### Identification of predictive biomarkers of response to treatments

To get more insights into molecular mechanisms of response and/or resistance observed with these treatments, we first analyzed the pharmacodynamics markers for each compound in order to confirm target engagement and pathway inhibition (Supplementary Figures S6 and S7). Treatment with AEB071 led to decreased phospho-PKCδ (pPKCδ) in all PDXs confirming activity of the compound and inhibition of the PKC pathway. In the three models that are sensitive to RAD001 (MM33, MM52, MP42), we observed a decreased in phospho-S6 (pS6) levels after RAD001 treatment, confirming that RAD001 blocked mTORC1 activity in these models. We did not observe such a decrease in the two non-responders to RAD001, MP46 and MP55 (Supplementary Figure S6). Thus, RAD001 failed to block mTORC1 activity in these two PDXs, which may explain their absence of response to RAD001. Given that CGM097 blocks protein-protein interaction between p53 and MDM2 and MDM2 is an E3 ubiquitin ligase that targets p53 for degradation by the proteasome, CGM097 treatment should lead to increased expression of p53, which in turn should result in increased expression of p21 (a direct transcriptional target of p53). This was indeed observed in all PDXs, confirming that CGM097 was active *in vivo* with the chosen dosage schedule (Supplementary Figure S7).

Next, we sought for biomarkers in various available sets of molecular data on our UM PDXs (untreated), namely gene mutations, gene expression profiles and Reverse-Phase Protein Array (RPPA)-based protein expression. Since no significant differential response among the models was observed for MEK162, CGM097 and their combinations with AEB071, we focused on the other treatments and combinations. Neither the mutation patterns (Supplementary Table S1) nor the analysis of gene expression profiles (Supplementary Figure S8) could identify a signature of response to the drugs. RPPA data allowed to identify a few potential proteins correlated to response (Supplementary Tables S7 and S8). They include PKCα for AEB071 and proteins of the PI3K/AKT/mTOR pathway in the combination therapy with RAD0001. These potential predictive biomarkers would need to be confirmed on a larger dataset including more models.

### The combination activities identified *in vivo* extend to uveal melanoma cell lines

In order to further understand the underlying mechanism of combination activity, the AEB071-based combinations with MEK162, RAD001 and CGM097 were tested in our panel of UM cell lines (Supplementary Table S9).

Each pairwise combination was tested at multiple concentrations using a five dose matrix. The dose range for each drug was adjusted according to preliminary experiments. AEB071, MEK162 and CGM097 were tested from 0 to 2μM, while the maximal dose used for RAD001 was 0.1μM. Since 92.1 and MM66 cells were highly sensitive to AEB071 treatment and had a much lower IC50 compared to the other cell lines, the top dose for AEB071 was set at 0.5μM in these two models. Synergy score and isobolograms were generated to quantify the combination strength using the Loewe algorithm. A synergy score higher than 2 was considered as significant (see Methods) [38].

The dose response curves for each drug used as a single agent are depicted in Supplementary Figures S9-S11. While AEB071, MEK162 and CGM097 showed varying degrees of growth inhibition depending on the cell line, ranging from 24-99% for the highest chosen dose, the maximal growth inhibitory effect obtained with RAD001 was approximately 50% as previously observed (Supplementary Table S10) [25]. Notably and as expected, AEB071 did not affect the proliferation of control cells that do not harbor *GNAQ/11*mutation.

All combination results are summarized in Supplementary Figures S12-S14. Importantly, for the three combinations, co-treatment of the two agents led to an enhanced anti-proliferative effect compared to the corresponding monotherapies for most of the tested doses and in the majority of *GNAQ/11* mutated models.

**Figure 3:**
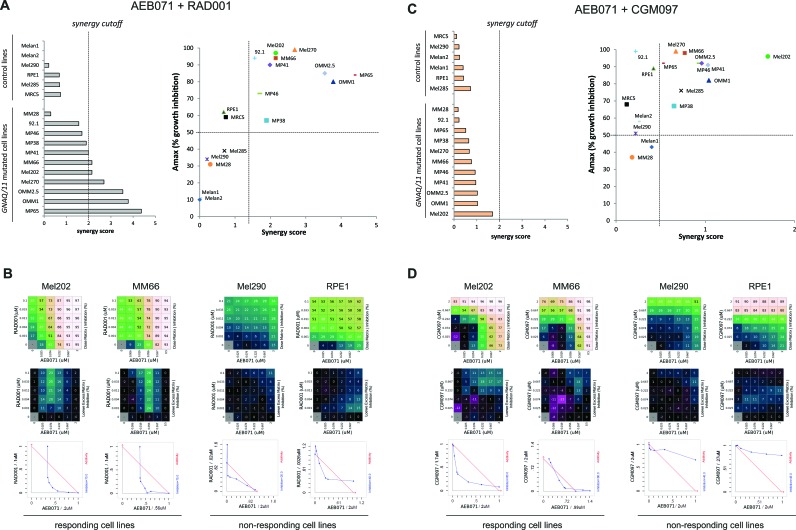
*In vitro* evaluation of AEB071 + RAD001 and AEB071 + CGM097 combinations **A.-B.** AEB071 + RAD001. **C.-D.** AEB071 + CGM097. **A.-C.** *Left:* histogram ranking all tested cell lines according to their synergy score. *Right:* Dot Plot representing Amax values (y-axis) and synergy scores (x-axis) for all tested cell lines. **B.-D.** Examples of two cell lines with combination activity (Mel202 and MM66) and two cell lines with no combination activity (Mel290 and RPE1). The matrix representing percentage of growth inhibition *(top panels)*, the matrix with the Loewe Excess results *(middle panels)* and isobolograms *(bottom panels)* are shown. In the isobolograms, the expected additivity line is in red and experimental data are represented in blue.

To determine to which extent co-inhibition of PKC with MEK1/2, mTORC1 or p53-MDM2 have combination activity in UM cell lines compared to control lines, results were represented as a dot plot showing, for each cell line, the synergy score and Amax value, with the Amax corresponding to the maximal growth inhibition effect seen in the combination treatment (Figure 3A and 3B; Supplementary Figure S15). We observed that the combination between of AEB071 and MEK162 or RAD001 were more synergistic compared to the combination between of AEB071 and CGM097. Indeed, AEB071 + MEK162 and AEB071 + RAD001 treatments resulted in synergy scores higher than 2 for 63.6 % (7 out of 11) and 54.5 % (6 out of 11) of *GNAQ/11* mutated UM cell lines respectively [39]. In contrast, AEB071 + CGM097 co-treatment gave rise to synergy scores around 1 for all *GNAQ/11* mutated UM models, indicating that this combination was rather additive. Results from two representative cell lines in which combination activity was observed (Mel202 and MM66) and two cell lines in which no combination activity was observed (Mel290 and RPE1) are presented in Figure 3C and 3D and Supplementary Figure S15B. It is important to note that, for both combinations, all *GNAQ/11* mutated cell lines, with the exception of the MM28 model, have overall higher synergy scores and Amax values compared to control lines. This observation highlights the value of these combinations for *GNAQ/11* mutated UM models and reveals a potential for achieving a therapeutic window using these drug combination approaches.

Even if the AEB071 + MEK162 association led to the highest synergy among our panel of cell lines, our *in vivo* experiments demonstrated that this combination was less efficient *in vivo* than AEB071 + RAD001 and AEB071 + CGM097 co-treatments. Indeed, taking all mice and all models together, 29% of mice had an ORR inferior to −0.75 after AEB071 + MEK162 treatment, whereas 49% and 79% of mice showed similar response under AEB071 + RAD001 and AEB071 + CGM097 associations respectively (Figure 1B and Supplementary Table S5). These results confirm the overall superior *in vivo* efficacy of AEB071 combined with RAD001 or CGM097.

### Co-inhibition of PKC and mTORC1 or PKC and p53-MDM2 leads to induction of apoptosis in most *GNAQ/11* mutated UM cell lines

**Figure 4:**
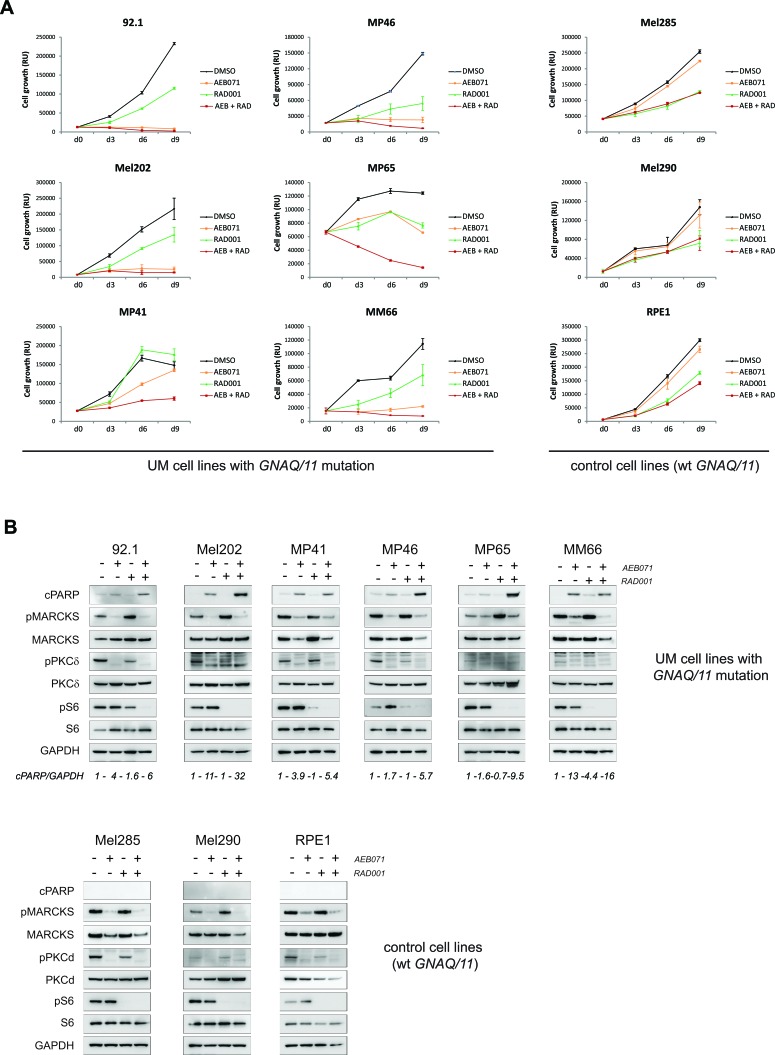
Co-inhibition of PKC and mTORC1 induces cell death in the majority of UM cell lines AEB071 (inhibition of PKC) and RAD001 (inhibition of mTORC1) were used respectively at 500 nM and 100 nM final concentration. **A.** Growth curve under treatment with AEB071 or/and RAD001. Cell viability was measured every 3 days with compound replacement at day 6. Averages between triplicates are represented ± SEM. **B.** Molecular analyses by western blot. Apoptosis was assessed by cPARP. pMARCKS and pPKCd were used as pharmacodynamic markers for AEB071 activity, while pS6 was used as the marker for RAD001 activity.

**Figure 5:**
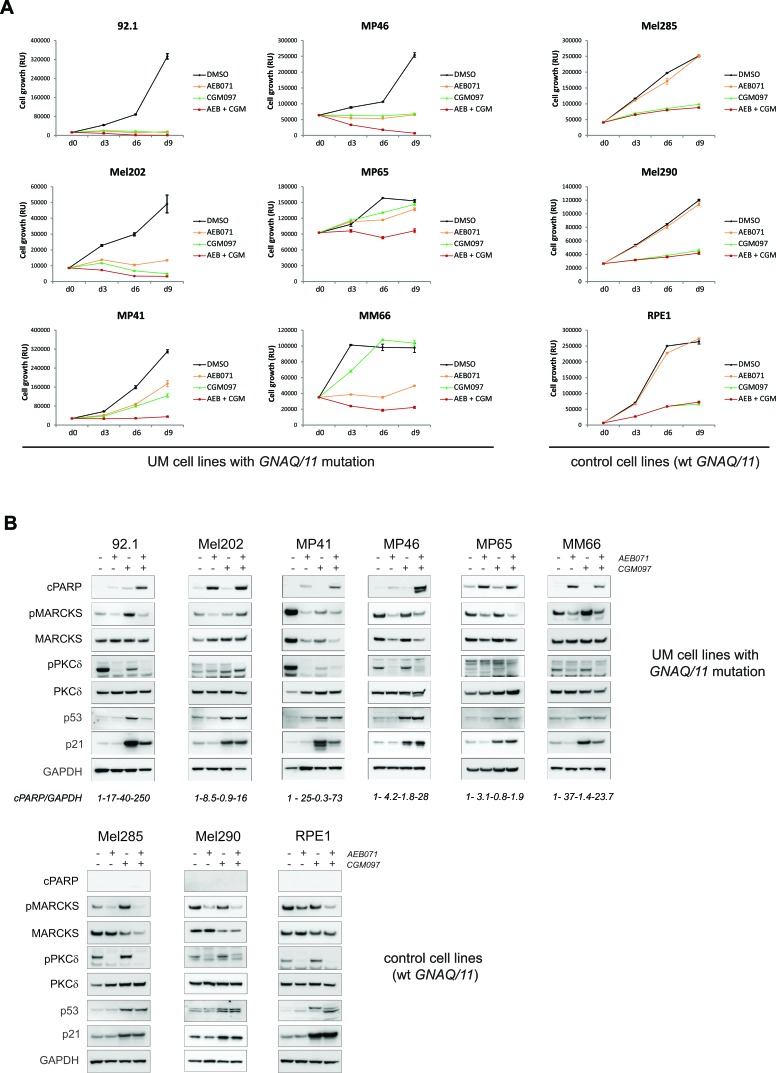
Co-inhibition of PKC and MDM2 induces cell death in the majority of UM cell lines AEB071 (inhibition of PKC) and CGM097 (inhibition of MDM2) were used respectively at 500 nM and 1 μM final concentration. **A.** Growth curve under treatment with AEB071 or/and CGM097. Cell viability was measured every 3 days with compound replacement at day 6. Averages between triplicates are represented ± SEM. **B.** Molecular analyses by western blot. Apoptosis was assessed by cPARP. pMARCKS and pPKCd were used as pharmacodynamic markers for AEB071 activity, while p53 and p21 were used as the marker for CGM097 activity.

To assess whether the two combinations that are the most efficient *in vivo* lead to growth arrest or apoptosis in our cell line models, we followed their growth during nine days of treatment with DMSO and each drug alone or in combination. Molecular studies were performed in parallel to confirm target engagement and pathway inhibition as well as to look for apoptosis (Figures 4 and 5; Supplementary Figures S16-S17).

We observed that co-treatment of AEB071 and RAD001 led to an enhanced loss of viability compared to monotherapies only in *GNAQ/11* mutated models. The combination effect was stronger in cell lines with lower responses to AEB071 used as a single agent (MP41, MP46, MP65, MM66). Overall, the combination treatment led to a loss of viability in all *GNAQ/11* mutated lines (Figure 4A and Supplementary Figure S16A). We next measured apoptosis using western blot analyses for cleaved PARP (cPARP) after three days of treatment (Figure 4B and Supplementary Figure S16B). In all of the six representative *GNAQ/11* mutated cell lines, AEB071 slightly induced cPARP. The combination with RAD001 led to a significantly stronger increase in cPARP in four out of six lines (92.1, Mel202, MP46, MP65), highlighting the enhanced apoptosis triggered by the combination treatment (see quantification of cPARP/GAPDH). In the other cell types for which cytostasis was observed in the proliferation assay, no induction of cPARP was detected (Supplementary Figure S16B). To get a more precise measurement of apoptosis, we performed AnnexinV staining. The induction of apoptosis by the combination treatment compared to monotherapies was confirmed in five of the eleven tested cell lines (MP46, MP65, MM66, Mel270, OMM2.5) (Supplementary Figures S18-S20). In conclusion, evidence of apoptosis (cleaved-PARP and/or AnnexinV staining) was observed in at least seven cell lines after three days of treatment. The effect of the co-inhibition of PKC and mTORC1 was less pronounced *in vitro* than *in vivo*, most likely due to the effect of RAD001 on non-tumor cells surrounding the tumor in *in vivo* settings.

Finally, target engagement and pathway inhibition were confirmed for both inhibitors (Figure 4B and Supplementary Figure S16B). Validation of AEB071 activity was done using the markers phosphorylated MARCKS (pMARCKS) and pPKCδ, while pS6 was used for RAD001. In all models, *GNAQ/11* mutated and wt, AEB071 treatment led to decreased pMARCKS and pPKCδ. Likewise, RAD001 treatment decreased the amount of pS6 in all models. Co-treatment with AEB071 and RAD001 did not alter the level of pathway inhibition seen with single agents.

Like the combination of AEB071 + RAD001, combination of AEB071 and CGM097 further blocked cell proliferation compared to single agent treatment in most *GNAQ/11* mutated cell lines (Figure 5A and Supplementary Figure S17A). The control lines did respond to CGM097 but not to AEB071, and their combination of both did not enhance the effect of CGM097 monotherapy in these models.

Effectiveness of each drug at the concentration used was verified using their corresponding pharmacodynamic markers. Treatment with AEB071 led to decreased pMARCKS and pPKCδ confirming activity of the compound and inhibition of the PKC pathway. Increased expression of p53 and its target p21 confirmed that 1μM of CGM097 was sufficient to inhibit MDM2 function and to activate the p53 pathway.

We next evaluate apoptosis by first looking at cPARP expression (Figure 5B and Supplementary Figure 17B). 92.1, Mel202, MP41, MP46 and Mel270 cells showed an increase of cPARP levels in the combination treatment compared to DMSO control and single agents. The MM66 cell line did not show an increase in cPARP, while a loss of viability under AEB071 + CGM097 treatment was observed in the growth curve assay. It is possible that the ATP release as a read-out of cell number or the time point used for the WB analyses were not appropriate to correlate both studies. AnnexinV staining was used to better quantify apoptosis and confirmed that the combination treatment strongly induced apoptosis in five cellular models (92.1, Mel202, MP46, MM66, Mel270) compared to DMSO or monotherapies (Supplementary Figures 21-23).

Interestingly, when comparing all cell lines together, the induction of apoptosis by AEB071 + CGM097 was much stronger than the one observed with AEB071 + RAD001 (see quantification of cPARP/GAPDH in Figures 4B and 5B; AnnexinV staining in Supplementary Figures S15-18 and S20-21), reinforcing our previous finding of the higher *in vivo* efficacy of AEB071 + CGM097 combination on AEB071 + RAD001 treatment.

In conclusion, our *in vitro* findings show that co-inhibition of PKC and mTORC1 or PKC and p53-MDM2 are effective combination strategies for *GNAQ/11* mutated UM models. Both co-treatments led to induction of apoptosis in most of the cellular models tested, with a stronger cell death observed with combination of PKC and p53-MDM2 inhibitors.

## DISCUSSION

About one third of UM patients develop metastases for which no curative treatment exists. Due to mutations in *GNAQ/11* genes and subsequent activation of the PKC pathway, the PKC inhibitor AEB071 has been tested in preclinical and clinical settings. Although treatment with AEB071 as a single agent resulted in tumor stasis in 50% of UM patients and a progression free survival of 15 weeks, our evolving hypothesis is that combination treatment including a PKCi will result in an improved clinical response. Taking advantage of our large panel of UM preclinical models, we have evaluated the efficacy of four AEB071-based combinations in five PDX models, with *in vitro* validation for the most promising approaches. Through this work, we have discovered two novel promising therapeutic strategies by co-targeting PKC and mTORC1 or PKC and p53-MDM2.

Based on our current knowledge of UM, we initially tested combinations targeting PKC with MEK1/2, CDK4/6, mTORC1 or p53-MDM2. Due to the hyperactivated MAPK pathway and the absence of sustained inhibition of MAPK signaling with PKCi alone, combination of PKCi + MEKi has been considered as a good therapeutic strategy. In our study, treatment of AEB071 + MEK162 showed good synergy in our panel of UM cellular models and led to a significant reduction of tumor growth in all PDXs, but without clear tumor stabilization or regression as opposed to what was described in UM xenograft models [16]. A multicenter phase II clinical trial was conducted to assess this combination in metastatic UM patients but was closed early due to toxicity issues. Optimization of dose and dosing regimen as well as the use of alternative MEKi or PKCi may further improve the therapeutic index and clinical benefit to patients of this combination. Because of high cyclin D1 and Rb expressions observed in UM [29][30][31], we evaluated the combination of PKCi + CDK4/6i using AEB071 and LEE011 compounds. Only a limited efficacy of LEE011 was observed and no clear evidence of increased efficacy combining it with AEB071 was detected, indicating that co-inhibition of PKC and CDK4/6 is not an optimal strategy for treating UM. The absence of increased efficacy after AEB071 + LEE011 combination could be in part explained by the fact that AEB071 decreases expression of cyclin D1 and Rb proteins and induces G1/S cell cycle arrest [16][17][40], effects that are specific to CDK4/6 targeting.

Two combinations appear as promising approaches in our *in vivo* combination screen. Dual inhibition of PKC and mTORC1 (AEB071 + RAD001) was able to significantly reduce tumor growth compared to each monotherapy, with two tumor regressions and one tumor stabilization observed. More strikingly, co-targeting of PKC and p53-MDM2 (AEB071 + CGM097) led to tumor stasis or regression in all five PDX models. Importantly, similar results were obtained using our panel of cell lines. Both combinations induced apoptosis or growth arrest in most *GNAQ/11* mutated cell lines, reinforcing the value of these combination regimens for UM.

Data mining of mutations, gene expressions and RPPA profiles of our PDXs could not allow the identification of predictive biomarkers of response. This is most probably due to the low number of models and the fact that, in some therapeutic situations, all or almost all models are responding to treatment (CGM097 and CGM097-based combinations). Clinical trials with tumor sample collection on a higher number of patients might allow the identification and the validation of such biomarkers.

To further understand the mechanism of combination activity, we evaluated the synergy between AEB071 + RAD001 and AEB071 + CGM097. We observed that, for most *GNAQ/11* mutated UM cell lines, the AEB071 + RAD001 combination was synergistic while AEB071 + CGM097 co-treatment was additive. We could make a similar conclusion using our scoring method *in vivo*, indicating that PKC and PI3K/AKT/mTOR pathway may be connected while the PKC and MDM2/p53 signaling may be independent molecular cascades. More in depth studies are required to understand these positive combination activities. In particular, the combination between AEB071 and BYL719 (a PI3Kα inhibitor) has shown some efficacy in preclinical studies [27]. To decipher if the mechanism of PKC + mTORC1 inhibition is similar than the one of PKC + PI3Kα, it would be interested to compare them with an association of PKCi with dual mTORC1/mTORC2 inhibitors. Our study also clearly demonstrates that an additive combination with a high combination Amax value could still be beneficial and can translate to *in vivo* tumor regressions, and thus should be taken into account when similar *in vitro* combination screens are performed.

Notably, our results were independent of BAP1 status. BAP1 is important for tumor progression; however some BAP1-positive patients still develop metastases, suggesting that other pathways are important to drive metastatic outgrowth. In contrast, the effects of the combinations were associated with *GNAQ/11* status, indicating that mutated GNAQ/11 proteins are likely to be UM drivers.

Our preclinical approach allows the identification of novel therapeutic approaches for UM patients and provides a solid preclinical package for a translation into clinical trials. This method should therefore be applied to test novel targets/compounds, such as molecules targeting the Hippo/YAP pathway, recently discovered as upregulated in UM [41].

In conclusion, we have shown that dual inhibition of PKC + p53-MDM2 and PKC + mTORC1 are more efficacious than other previously tested combinations in UM, with striking tumor regression observed in several *in vivo* models after co-targeting of PKC and p53-MDM2. Moreover, our study provides a strong rationale to test these combinations in the clinical setting for metastatic UM patients.

## MATERIALS AND METHODS

### Uveal melanoma preclinical models

Five PDXs representative of the UM disease were used: MP42, MP46, MP55, MM33 and MM52 (Supplementary Table S1). The main molecular features of these PDXs have been described in [35][36].

Fifteen *in vitro* cellular models, isolated either from primary tumors or metastases, were used (Supplementary Table S9). MP38, MP41, MP46, MP65, MM28 and MM66 cell lines were established in our laboratory as described in [26]. These cell lines were characterized by Short Tandem Repeat (STR) Method (Promega, GenePrint 10 System). 92.1 and Mel202 cell lines were purchased from The European Searchable Tumour Line Database (Tubingen University, Germany); MRC5 and RPE1 lines from ATCC. OMM1, OMM2.5, Mel285 and Mel290 cells were kindly provided by P.A. Van Der Velden (Leiden University, The Netherlands) and genotyped as described in [26]. Two primary cultures of normal melanocytes isolated from a human choroid were kindly given by G. Liot (Institut Curie, France). All culture conditions are described in the Supplementary Materials.

### Compounds

All drugs used in this study (AEB071, CGM097, RAD001, MEK162, LEE011) were obtained from Novartis Institutes for Biomedical Research (NIBR, Cambridge, USA). AEB071, MEK162, RAD001, CGM097 and LEE011 are selective inhibitors of PKC, MEK1/2, mTORC1, MDM2 and CDK4/6 respectively. For *in vivo* administration, compounds were diluted in 20% propylene glycol + 50% solutol + 30% PBS. The control groups were treated with this solution (vehicle). Each compound was administrated 5 days a week at the dose indicated in each figure legend (see Supplementary Materials for further details).

For *in vitro* experiments, compound powders were dissolved in DMSO for a stock concentration of 10mM final, aliquoted and stored at −20°C. Further dilutions were made according to each experiment design.

### *In vivo* drug testing experiments

Tumor fragments of 30-60mm^3^ were grafted subcutaneously into the interscapular fat pad of four to six week-old mice. When tumors reached a size of about 50-150mm^3^, mice were randomly assigned to control or treatment groups. Between six to nine mice per group were included in each experiment. Mice were sacrificed when their tumor reached a volume of 2500mm^3^. There were two sets of *in vivo* independent experiments: a first one testing dose-dependent efficacy of AEB071 administered alone, and a second one testing AEB071 (120 mg/kg) alone and in combinations (AEB071+RAD001/CGM097/MEK162/LEE011). Hence, in this second set of experiments, control groups are similar in each model for all tested combinations, and the doses of each compound were identical in all tested combinations and in each model.

Tumor growth was evaluated using standard methods and is described in the Supplementary Materials.

Studies have been performed in compliance with the recommendations of the French Ethical Committee and under the supervision of authorized investigators. The experimental protocol and animal housing followed institutional guidelines as put forth by the French Ethical Committee (Agreement C75-05 −18, France) and the ethics committee of Institut Curie.

### Statistical tests for *in vivo* experiments

Two by two comparison of TGI observed in two arms was done using a two-sided Mann-Whitney test based on the RTVV. For all pairwise comparisons based on the proportions of tumors with a particular RTV or ORR, a two-tailed Fisher's exact test was used. All statistical tests were carried out bilaterally calculating two-tailed p-values. Results were considered statistically significant when p ≤ 0.05 (95% confidence interval).

### Drug combination cell viability assay

Cells were seeded at appropriate concentration in three 96-well plates following a 6×6 matrix design. The day after, each drug was added following a matrix dilution format. 1:3 serial dilutions were tested to result in a total of six serial dilutions, including the DMSO control. Cell viability was measured after five days of drug treatment using the MTT assay (Sigma). Results were read using a spectrophotometer, and expressed as relative percentages of metabolically inactive cells compared with DMSO-treated controls (percentage of growth inhibition). All different combinations were tested on the whole cell line panel for each experimental procedure. The tests were repeated until at least an independent duplicate for each drug combination was obtained.

### Evaluation of *in vitro* combination activity

Combination effects were calculated with the Combination Analysis Module software, a Novartis in-house software application which implements the full set of combination analysis methods as described by Lehar et al. [38][42]. A weighted “Synergy Score” was calculated across the dose matrix that adjusts for dose sampling and coverage and weights to favor combination effects at high inhibition levels. A synergy score higher than 2 was considered as significant when compared to the variation of synergy scores seen within self-crosses (drug-with-self; theoretical synergy score of 0).

### Growth curve assay and evaluation of loss of viability

At day 0, cells were plated in triplicate at appropriate concentration in 96-well plates. Four conditions were tested for each cell line: DMSO, Drug A, Drug B and Drug A+B. At day 1, each drug was added to each well. Optimal drug concentrations were chosen from the combination experiments: AEB071 was used at 500nM, RAD001 at 100nM and CGM097 at 1μM. The amount of DMSO was adjusted to 0.2% in each mix to get the same percentage of DMSO for each treatment condition. Compounds were replenished at day 6. At day 3, day 6 and day 9, viability was measured using the CellTiterGlo assay (Promega). The average between triplicates was made and represented ± SEM. All cell lines were tested at the same time and at least two independent experiments were performed to confirm results reproducibility.

### Western blot analyses

Cells were cultured in 10 cm-diameter dishes and treated with DMSO or each drug as single agent or combination for 72h. Western blot analyses were performed using standard procedures. GAPDH was used for normalization between samples. Primary antibodies were diluted in TBST + 0.5% BSA at an appropriate dilution and incubated overnight at 4°C. All antibodies used in this study are listed in Supplementary Materials. Signal was detected using secondary antibodies coupled with HRP (Jackson laboratory). Luminescent signal was detected using a LAS-3000 Luminescent Image analyzer. Four tumors per therapeutic group have been analyzed.

## SUPPLEMENTARY MATERIALS FIGURES AND TABLES


