# Supplementary material for: Dual inhibition of protein kinase C and p53-MDM2 or PKC and mTORC1 are novel efficient therapeutic approaches for uveal melanoma
Source: Oncotarget. 2016 May 22;7(23):33542–56. doi: 10.18632/oncotarget.9552 (PMC5085101; doi:10.18632/oncotarget.9552)
Supplement: Supplementary file 1 [file oncotarget-07-33542-s001.pdf]

Dual inhibition of protein kinase C and p53-MDM2 or PKC and mTORC1 are novel efficient therapeutic approaches for uveal melanoma

Supplementary Data

Supplementary Materials:

Evaluation of tumor growth of *in vivo* experiments

Dosage schedule for *in vivo* experiments

Gene expression analyses

RPPA analyses

List of antibodies used in the study for western blot analyses.

Supplementary Tables:

Table S1: Biological characteristics of the five UM PDXs used in the study.

Table S2: *In vivo* efficacy of AEB071 at the doses 120mg/kg/day and 240mg/kg/day.

Table S3: *In vivo* efficacy of targeted therapies administered alone.

Table S4: *In vivo* score of efficacy of targeted therapies administered alone or in AEB071-based combinations.

Table S5: *In vivo* efficacy of AEB071-based combinations.

Table S6: p values of combinations versus monotherapy treatments *in vivo*.

Table S7: List of the proteins studied by RPPA.

Table S8: predictive protein markers for *in vivo* response.

Table S9: Genetic status of the known altered genes in UM for all cell lines used in this study.

Table S10: Amax values and synergy scores in all cell lines.

Figure S1: *In vivo* efficacy of monotherapies in UM PDXs.

Figure S2: Overall response rate of all in *in vivo* tested monotherapies.

Figure S3: Overall response rate of AEB071 + RAD001 (A) and AEB071 + CGM097 (B).

Figure S4: *In vivo* efficacy of AEB071 + MEK162 combination in UM PDXs.

Figure S5: *In vivo* efficacy of AEB071 + LEE011 combination in UM PDXs.

Figure S6: Pharmacodynamic marker analysis for the AEB071 + RAD001 combination.

Figure S7: Pharmacodynamic marker analysis for the AEB071 + CGM097 combination.

Figure S8: Gene expression levels in UM PDXs and corresponding patients.

Figure S9: Dose response curves to AEB071, RAD001 and CGM097 in the *GNAQ/11* mutated UM cell lines used in this study

Figure S10: Dose response curves to AEB071, RAD001 and CGM097 in control cell lines

Figure S11: Dose response curves to MEK162 in the *GNAQ/11* mutated UM cell lines and control lines

Figure S12: *In vitro* evaluation of AEB071 + RAD001 combination.

Figure S13: *In vitro* evaluation of AEB071 + CGM097 combination.

Figure S14: *In vitro* evaluation of AEB071 + MEK162 combination.

Figure S15: *In vitro* evaluation of AEB071 + MEK162 combination - continued

Figure S16: Co-inhibition of PKC and mTORC1 induces cell death in the majority of UM cell lines.

Figure S17: Co-inhibition of PKC and MDM2 induces cell death in the majority of UM cell lines.

Figures S18, S19, S20: Quantification of apoptosis by Annexin V after PKC and mTORC1 inhibition alone or in combination.

Figures S21, S22, S23: Quantification of apoptosis by Annexin V after PKC and mTORC1 inhibition alone or in combination.

SUPPLEMENTARY MATERIALS

Evaluation of tumor growth of *in vivo* experiments

Tumor growth was evaluated by measuring with a caliper two perpendicular tumor diameters twice a week. Individual tumor volume, relative tumor volume (RTV) and tumor growth inhibition (TGI) were calculated according to a standard method (35). Tumor stability or shrinkage was defined as a RTV $\leq$ 1 at the end of experiments. To evaluate the response to each treatment according to individual mouse variability, we have considered each mouse as one tumor-bearing entity. We have defined a relative tumor volume variation (RTVV) of each treated mouse: RTVV=Vt/Vc, where Vt is the volume of the treated mouse and Vc the median tumor volume of the corresponding control group, at the end of treatment. For each mouse, we calculated an overall response rate (ORR) using the formula: ORR=[(RTVV)-1]. A tumor was considered as responding to therapy when ORR was lower than -0.5. Since data were normalized to each control group, results of independent *in vivo* experiments could be merged. The main interest of the ORR consists of the possibility to combine various models in a same representation due to the normalization performed for each treated PDX and therefore, to allow statistical comparisons in all models included in the experimental program.

Dosage schedule for i*n vivo* experiments

AEB071 was administered *per os* twice a day, 5 days/week at a daily dose of 120 or 240 mg/kg according to the *in vivo* experiment design. MEK162 was administered *per os* twice a day, 5 days/week at a daily dose of 3.5mg/kg. RAD001 was administered *per os*, 5 days/week at a daily dose of 5mg/kg. CGM097 was administered *per os*, 5 days/week at a daily dose of 100mg/kg. LEE011 was administered *per os*, 5 days/week at a daily dose of 75mg/kg. All these doses have been defined based on previous preclinical data. In particular, as RAD001 has been reported at daily doses ranging from 1 (1, 2) to 10 mg/kg/day (3), and

because of the absence of toxicity when RAD001 was administrated alone and in combination with various other compounds using a dosage of 5 mg/kg per day, we have decided to maintain this schedule in order to perform the efficacy studies in our UM PDXs. Similarly, the dose of CGM097 was defined according to previous studies performed by Novartis, showing that a dose of 100 mg/kg appears to be well tolerated and efficacious in multiple xenografts studies, and leads to an exposure that is currently observed in patients (Novartis unpublished data).

There were two sets of *in vivo* independent experiments: a first one testing dose-dependent efficacy of AEB071 administered alone, and a second one testing AEB071 (120 mg/kg) alone and in combinations (AEB071+RAD001/CGM097/MEK162/LEE011). Hence, in this second set of experiments, control groups are similar in each model for all tested combinations, and the doses of each compound were identical in all tested combinations and in each model.

Gene expression analyses

Transcriptome profiling of patient tumors and corresponding PDXs was defined using Affymetrix Human Exon 1.0 ST array. RNA isolation, preparation, hybridization and raw data were obtained as previously described (Laurent C et al 36). Briefly, gene expression levels were estimated using custom Brainarray CDF (version 13) based on EntrezGene database. Data were summarized and normalized using RMA method. Raw and normalized data are publicly available on GEO repository under the accession number GSE78033 (http://www.ncbi.nlm.nih.gov/geo/query/acc.cgi?acc=GSE78033).

Transcriptome profiling of each studied PDX model at passage 1 were studied. Among these 5 samples, 500 most variant genes based on IQR measure (Inter Quartile Range) were selected. Clusters of samples based on these most informative genes were identified by hierarchical clustering using Pearson's correlation as metric to measure the distance between observations and Ward's method as linkage criterion to measure the distance between sets of observations.

RPPA analyses

An untreated tumor sample was collected for each model and from similar *in vivo* passages between them. These samples were analyzed for the normalized expression level of 93 proteins or phospho-proteins by RPPA as described in *Laurent et al., 2013*. The list of antibodies used in this study is shown in Supplementary Table S7. The five models were classified as low or high responders for each treatment. We performed pairwise comparisons by t-tests (two sided, 95% confidence intervals) between the both response groups for each treatment when it was possible (presence of a differential response and at least two models per group) and for each protein. All significant results are summarized in Supplementary Table S8.

List of antibodies used in western blot analyses.

**Protein target**	**Supplier and catalogue number**
GAPDH	Millipore #MAB374
Total MARCKS	CST #5607S
Phospho-MARCKS (Ser159/163)	CST #11992S
Total PKCδ	CST #9616S
Phospho-PKCδ (S299)	Abcam #ab133456
Total S6	CST #2317S
Phospho-S6 (S235/236)	CST #2211S
p53	CST #9282S
p21 Waf1/Cip1	CST #2947S
Cleaved PARP	Abcam #ab32561

AnnexinV/PI staining

For apoptosis evaluation, cells were harvested after 72h of treatment. Apoptosis was measured using the AnnexinV-FLUOS staining kit (Roche) according to the manufacturer's instructions. After sequential staining by AnnexinV and PI, flow cytometry analyses were performed on a LSRII Instrument (Becton Dickinson) using the FlowJo software. The percentages of living cells (low AnnexinV and low PI), apoptotic cells (high AnnexinV and low PI) and necrotic cells (high AnnexinV and high PI) were evaluated. Two to three independent experiments were performed and statistical analyses were made using two-way ANOVA test with Bonferroni correction.

SUPPLEMENTARY TABLES

Table S1: Biological characteristics of the five UM PDXs used in the study

PDXs	Origin	Histology	L3	*GNAQ*	*GNA11*	*BAP1*	*SF3B1*	Other mutations
**MP42**	Primary tumor	Spindle cells	0	wt	mut	mut	wt	/
**MP46**	Primary tumor	Mixed cells°	+	mut	wt	wt	wt	*Myc*
**MP55**	Primary tumor	Epithelioid	+	wt	mut	mut	wt	/
**MM33**	Skin metastase	Epithelioid	0	mut	wt	wt	wt	*TSC2*
**MM52**	Liver metastase	Mixed cells°	+	mut	wt	mut	mut	/

Abbreviations: L3, monodisomy or isodisomy of the chromosome 3; Mixed cells included epithelioid and spindle cells.

Table S2: *In vivo* efficacy of AEB071 at the doses 120mg/kg/day and 240mg/kg/day
TGI (%): percentage of tumor growth inhibition
RTV ≤ 1 (%): percentage of mice with a RTV≤ 1
ORR (%): percentage of mice with a ORR either ≤ -0.5 or ≤-0.75

**PDXs**	**TGI (%)**			**RTV ≤ 1 (%)**			**ORR (%)**					
							**≤ -0.5**			**≤ -0.75**		
	**120**	**240**	**p**	**120**	**240**	**p**	**120**	**240**	**p**	**120**	**240**	**p**
**MP42**	64	83	0.003	25	100	0.007	75	100	0.467	12.5	75	0.041
**MP46**	61	84	< 0.001	0	62.5	0.026	87.5	100	1.000	0	100	< 0.001
**MP55**	16	47	0.001	0	0	1.000	0	12.5	1.000	0	0	1.000
**MM33**	0	42	0.015	0	0	1.000	0	0	1.000	0	0	1.000
**MM52**	20	41	0.078	0	33	0.206	0	33	0.206	0	11	1.000
**All**	/	/	/	**5**	**39**	**< 0.001**	**33**	**49**	**0.180**	**3**	**37**	**< 0.001**

Table S3: ***In vivo*** **efficacy of targeted therapies administered alone**

PDXs	TGI (%)				
	AEB071	MEK162	RAD001	CGM097	LEE011
MP42	77	36	29	56	30
MP46	30	23	12	59	/
MP55	7	13	14	70	/
MM33	24	35	70	56	33
MM52	34	50	71	70	44
**PDXs**	**RTV ≤ 1 (%)**				
	**AEB071**	**MEK162**	**RAD001**	**CGM097**	**LEE011**
**MP42**	86	0	29	29	0
**MP46**	0	0	0	0	/
**MP55**	0	0	0	0	/
**MM33**	0	0	25	0	0
**MM52**	0	12.5	50	37.5	0
**All**	**15**	**2.5**	**20**	**12.5**	**0**
**PDXs**	**ORR < -0.5 (%)**				
	**AEB071**	**MEK162**	**RAD001**	**CGM097**	**LEE011**
**MP42**	100	33	43	86	29
**MP46**	22	33	0	67	/
**MP55**	11	11	0	89	/
**MM33**	0	0	62.5	29	0
**MM52**	37.5	37.5	75	75	12.5
**All**	**32**	**22.5**	**34**	**70**	**13**
**PDXs**	**ORR < -0.75 (%)**				
	**AEB071**	**MEK162**	**RAD001**	**CGM097**	**LEE011**
**MP42**	43	0	14	14	0
**MP46**	0	11	0	22	/
**MP55**	11	0	0	44	/
**MM33**	0	0	25	0	0
**MM52**	0	0	25	25	0
**All**	**10**	**2.5**	**12**	**22.5**	**0**

Table S4: *In vivo* score of efficacy of targeted therapies administered alone or in AEB071-based combinations

A = AEB071; M = MEK162; R = RAD001; C = CGM097

Treatments		MP42	MP46	MP55	MM33	MM52	Total
4 monotherapies (score 1 to 4)	AEB071	1	2	4	4	4	**15**
	MEK162	3	3	2	3	3	**14**
	RAD001	4	4	3	1	1	**13**
	CGM097	2	1	1	2	2	**8**
3 combinations (score 1 to 3)	A + M	3	2	2	3	3	**13**
	A + R	1	3	3	1	1	**9**
	A + C	2	1	1	2	2	**8**

Table S5: *In vivo* efficacy of AEB071-based combinations

PDXs	TGI (%)			RTV ≤ 1 (%)			ORR (%)					
							≤ -0.5			≤ -0.75		
**Treatments**	**A**	**M**	**A+M**	**A**	**M**	**A+M**	**A**	**M**	**A+M**	**A**	**M**	**A+M**
**MP42°**	77	36	92	86	0	100	100	33	100	43	0	100
**MP46**	30	23	70	0	0	0	22	33	89	0	11	56
**MP55**	7	13	52	0	0	0	11	11	56	11	0	0
**MM33**	24	35	53	0	0	0	0	0	12.5	0	0	0
**MM52**	34	50	45	0	12.5	0	37.5	37.5	50	0	0	0
**All**	**/**	**/**	**/**	**15**	**2.5**	**17**	**32**	**22.5**	**61**	**10**	**2.5**	**29**
**Treatments**	**A**	**R**	**A+R**	**A**	**R**	**A+R**	**A**	**R**	**A+R**	**A**	**R**	**A+R**
**MP42°**	77	29	98	86	29	100	100	43	100	43	14	100
**MP46**	30	12	33	0	0	0	22	0	44	0	0	0
**MP55**	7	14	21	0	0	0	11	0	0	11	0	0
**MM33**	24	70	91	0	25	100	0	62.5	100	0	25	100
**MM52**	34	71	80	0	50	100	37.5	75	100	0	25	62.5
**All**	**/**	**/**	**/**	**15**	**20**	**56**	**32**	**34**	**66**	**10**	**12**	**49**
**Treatments**	**A**	**C**	**A+C**	**A**	**C**	**A+C**	**A**	**C**	**A+C**	**A**	**C**	**A+C**
**MP42°**	77	56	96	86	29	100	100	86	100	43	14	100
**MP46**	30	59	93	0	0	89	22	67	100	0	22	100
**MP55**	7	70	84	0	0	11	11	89	100	11	44	100
**MM33**	24	56	82	0	0	62.5	0	29	100	0	0	62.5
**MM52**	34	70	71	0	37.5	67	37.5	75	67	0	25	17
**All**	**/**	**/**	**/**	**15**	**12.5**	**64**	**32**	**70**	**95**	**10**	**22.5**	**79**
**Treatments**	**A**	**L**	**A+L**	**A**	**L**	**A+L**	**A**	**L**	**A+L**	**A**	**L**	**A+L**
**MP42°**	77	30	94	86	0	100	100	29	100	43	0	100
**MM33**	24	33	39	0	0	0	0	0	14	0	0	0
**MM52**	34	44	50	0	0	0	37.5	12.5	37.5	0	0	0
**All**	**/**	**/**	**/**	**15**	**0**	**32**	**32**	**13**	**50**	**10**	**0**	**32**

° All combinations induced complete remissions, 1/7 after AEB071 + MEK162 (14%), 4/7 after AEB071 + RAD001 (57%), 2/7 after AEB071 + CGM097 (29%), and 3/7 after AEB071 + LEE011 (43%).

Table S6: p-values of combinations *versus* monotherapy treatments *in vivo*

See pdf file

p-values were calculated for each two by two treatment comparisons. Each comparison was done between the treatments indicated in the columns B and C. Significant differences are highlighted in red.

Table S7: List of antibodies used with RPPA and corresponding targeted proteins

See pdf file

In total, we selected 93 proteins or phospho-proteins (center column) belonging to 18 signaling pathways or functions (left column) frequently altered in cancer. The supplier and reference of each antibody used in this RPPA study are specified in the right column.

Table S8: Predictive protein markers for *in vivo* response

Treatment(s)	Protein	Expression in	
		**sensitive models**	**resistant models**
**AEB071 (dose effect) or AEB071 + MEK162**<br>MP42, MP46 *vs* MP55, MM33, MM52	PKC α	+	-
**RAD001**<br>MM33, MM52 *vs* MP42, MP46, MP55	No difference between the both groups		
**AEB071 + RAD001**<br>MP42, MM33, MM52 *vs* MP46, MP55	STAT3	-	+
	p70 S6 Kinase	-	+
	Phospho-PTEN (ser 380/thr 382/383)	-	+

Table S9: Genetic status of the known altered genes in UM for all cell lines used in this study.

P (primary tumor), M (metastasis), – (wild type), + (mutant), N/A (non-applicable)

Tissue	Cell line name	Tumor	GNAQ mutation	GNA11 mutation	BAP1 mutation	BAP1 loss	SF3B1 mutation	eIF1AX mutation	Additional comments
**Uveal melanoma**	MP38	P	+	-	+	+	-	-	
	MP41	P	-	+	-	-	-	-	
	MP46	P	+	-	-	+	-	-	
	MP65	P	-	+	+	+	-	-	
	MM28	M	-	+	+	+	-	-	Liver metastasis
	MM66	M	-	+	-	-	-	-	Liver metastasis
	92.1	P	+	-	-	-	-	+	
	Mel202	P	+	-	-	-	+	-	
	Mel270	P	+	-	-	-	-	-	
	OMM1	M	-	+	-	-	-	-	Subcutaneous metastasis
	OMM2.5	M	+	-	-	-	-	-	Liver metastasis
	Mel285	P	-	-	-	-	-	-	
	Mel290	P	-	-	-	-	-	-	
**Normal choroid**	Melan-1	N/A	-	-	-	-	-	-	
	Melan-2	N/A	-	-	-	-	-	-	
**Normal retina**	RPE1	N/A	-	-	-	-	-	-	Immortalized with hTERT
**Normal lung**	MRC5	N/A	-	-	-	-	-	-	fibroblasts

Table S10: Amax values and synergy scores in all cell lines

This table corresponds to the results shows in Figures 3A and 3B and Supplementary Figure S15.

Amax values were extracted from the combination matrices. For AEB071, the average across tested combinations is shown.

	**Amax values (% growth inhibition)**						
**Cell line name**	**AEB071 2μM**	**MEK162 2μM**	**RAD001 0.1μM**	**CGM097 2μM**	**AEB071+ MEK162**	**AEB071+ RAD001**	**AEB071+ CGM097**
**MP38**	46	48	19	73	74	57	67
**MP41**	85	69	27	69	93	90	92
**MP46**	60	46	27	64	77	73	92
**MP65**	58.5	55	33	64	89	84	92
**MM28**	33	24	20	36	34	31	37
**MM66**	92 *(0.5μM)*	82	46	74	98	94	98
**92.1**	94.5 *(0.5μM)*	81	39	90	98	94	99
**Mel202**	84	84	45	83	97	97	96
**Mel270**	99	99	45	79	100	99	99
**OMM1**	47	57	52	73	77	80	82
**OMM2.5**	79	44	34	24	84	85	91
**Mel285**	14.5	35	35	72	35	39	76
**Mel290**	16.5	0	21	45	19	34	51
**Melan1**	8.5	-20	10	35	21	10	43
**Melan2**	-4	n/a	8	49	n/a	11	58
**RPE1**	23	74	54	91	78	62	89
**MRC5**	8	54	57	70	47	59	68

	Synergy scores		
**Cell line name**	**AEB071+ MEK162**	**AEB071+ RAD001**	**AEB071+ CGM097**
**MP38**	3.96	1.89	0.65
**MP41**	2.79	1.99	0.96
**MP46**	1.63	1.70	0.93
**MP65**	3.25	4.37	0.52
**MM28**	0.17	0.30	0.18
**MM66**	2.66	2.15	0.77
**92.1**	2.87	1.56	0.22
**Mel202**	3.51	2.15	1.70
**Mel270**	1.01	2.69	0.67
**OMM1**	3.94	3.78	1.04
**OMM2.5**	1.91	3.54	1.03
**Mel285**	0.48	0.70	0.73
**Mel290**	0.02	0.21	0.22
**Melan1**	0.02	0	0.40
**Melan2**	n/a	0.004	0.25
**RPE1**	1.43	0.68	0.42
**MRC5**	0.25	0.74	0.12

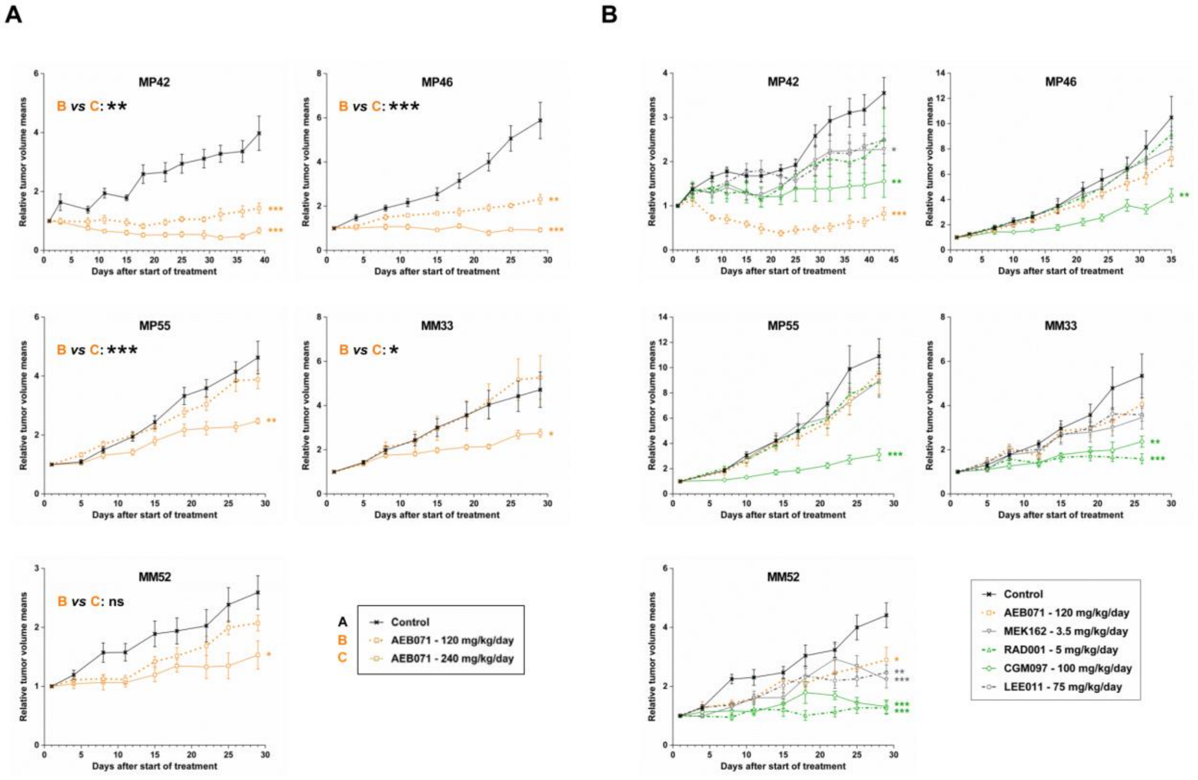

Figure S1: *In vivo* efficacy of monotherapies in UM PDXs. (A) Dose-dependent efficacy of AEB071. **(B)** Compared efficacy of AEB071, MEK162, RAD001, LEE011 and CGM097 treatment. Doses are indicated in the legend. Tumor growth is represented using the mean of the RTV (relative tumor volume) ± SD per group. *: p<0.05; **: p<0.01; ***: p<0.001.

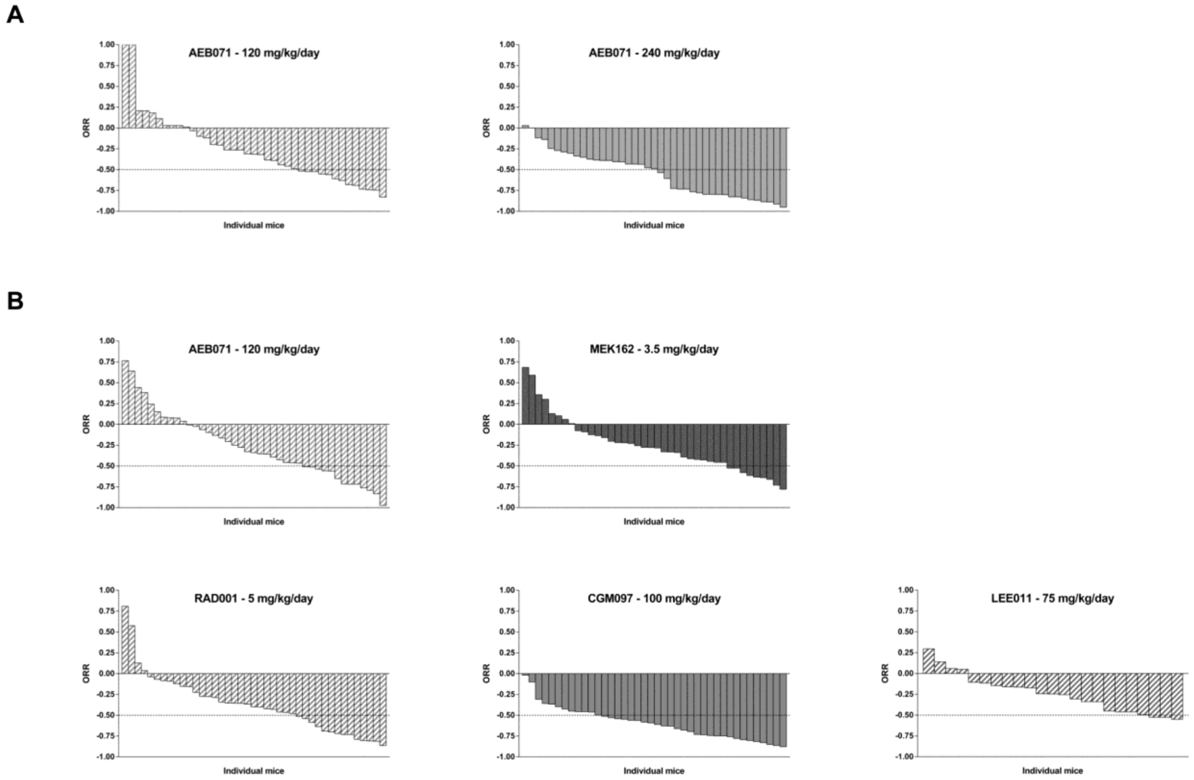

Figure S2: Overall response rate of all *in vivo tested* monotherapies. The overall response rate (ORR) of mice treated with X was defined as the relative tumor volume variation (RTVV) of each X-treated mouse calculated from the following formula: [(Vt/Vc) – 1], where Vt is the volume of the treated mouse and Vc the median volume of the corresponding control group at a time corresponding to the end of treatment. **(A)** ORR graphs of the dose-response experiment for AEB071 shown in Fig. S1A. **(B)** ORR graphs for each monotherapy, represented in Fig. S1B.

A

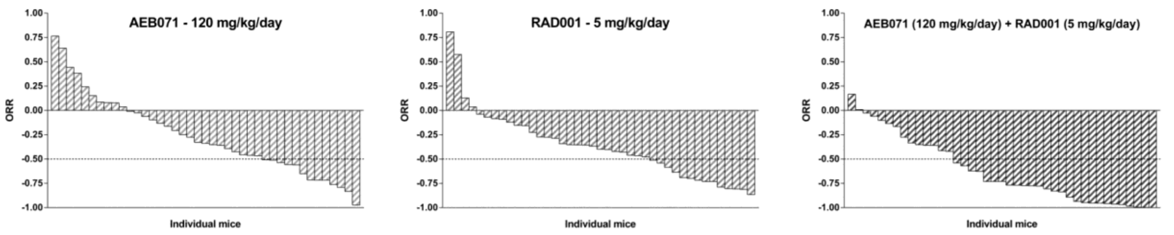

B

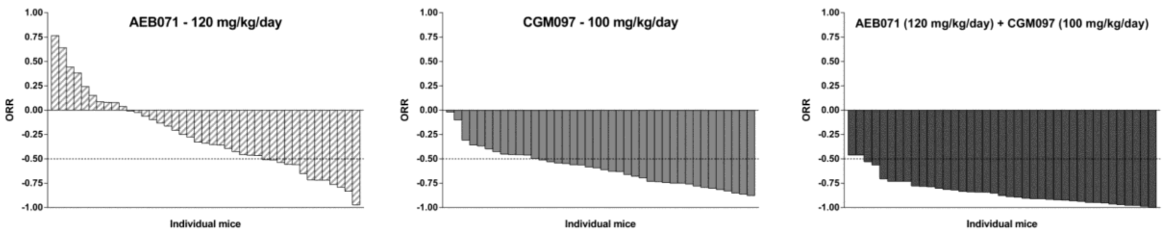

Figure S3: Overall response rate of AEB071 + RAD001 and AEB071 + CGM097. (A) ORR graphs of the AEB071 + RAD001 combination and each corresponding monotherapies. **(B)** ORR graphs of the AEB071 + CGM097 combination and each corresponding monotherapies.

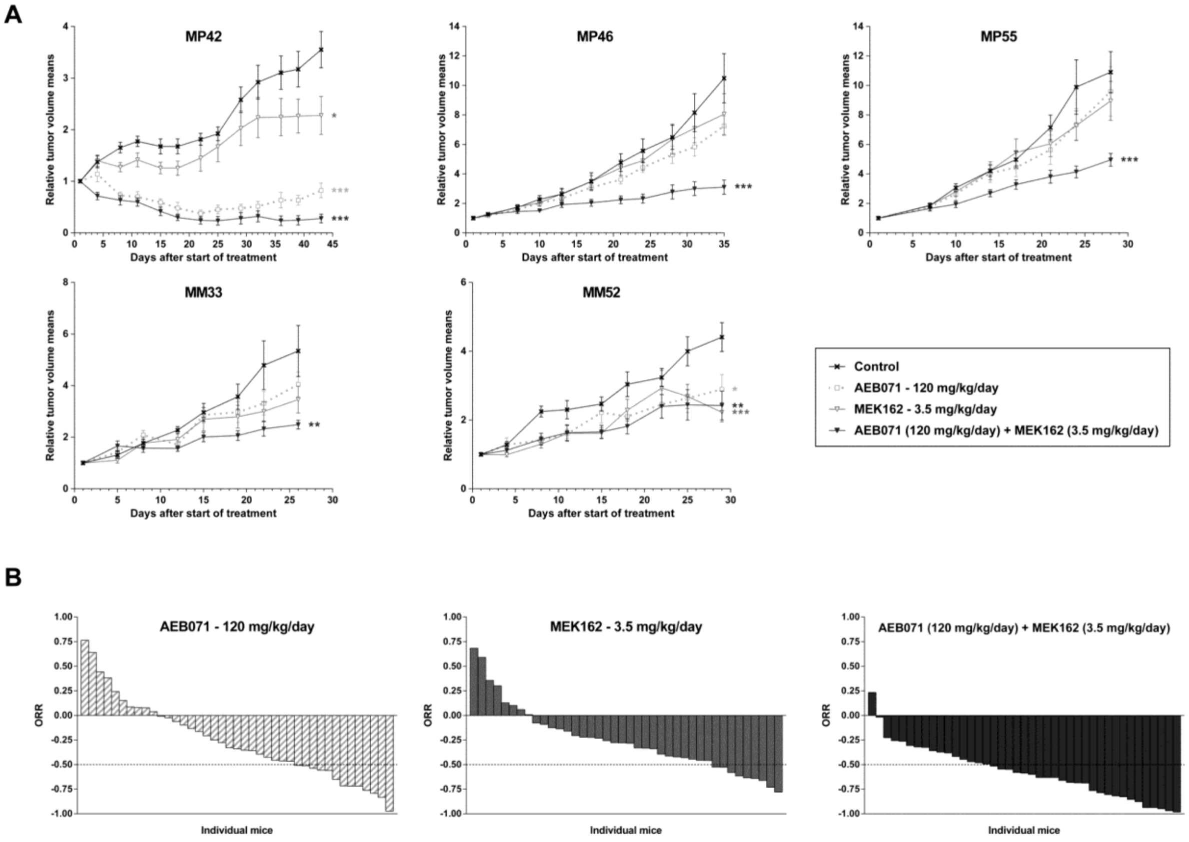

Figure S4: *In vivo* efficacy of AEB071 + MEK162 combination in UM PDXs. (A) Tumor growth curves, as mean of the RTV (relative tumor volume) ± SD. **(B)** ORR graphs.

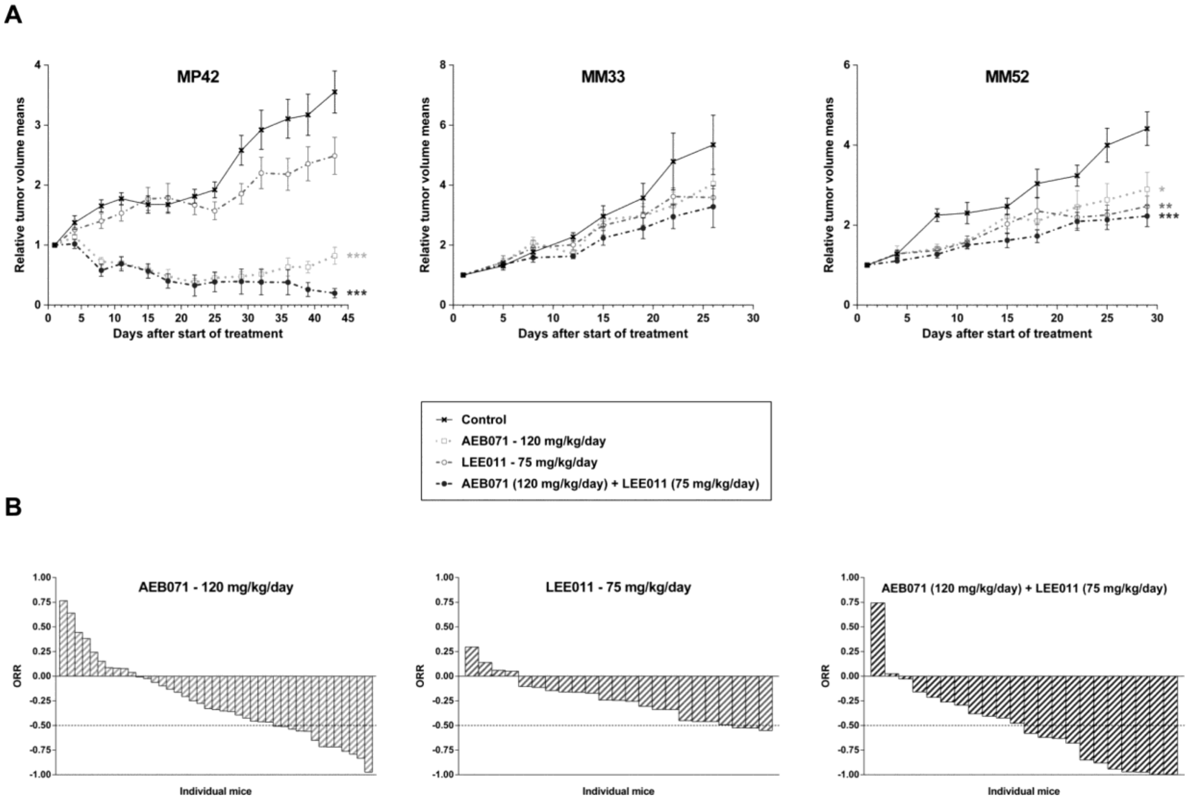

Figure S5: *In vivo* efficacy of AEB071 + LEE011 combination in UM PDXs. (A) Tumor growth curves, as mean of the RTV (relative tumor volume) ± SD. **(B)** ORR graphs.

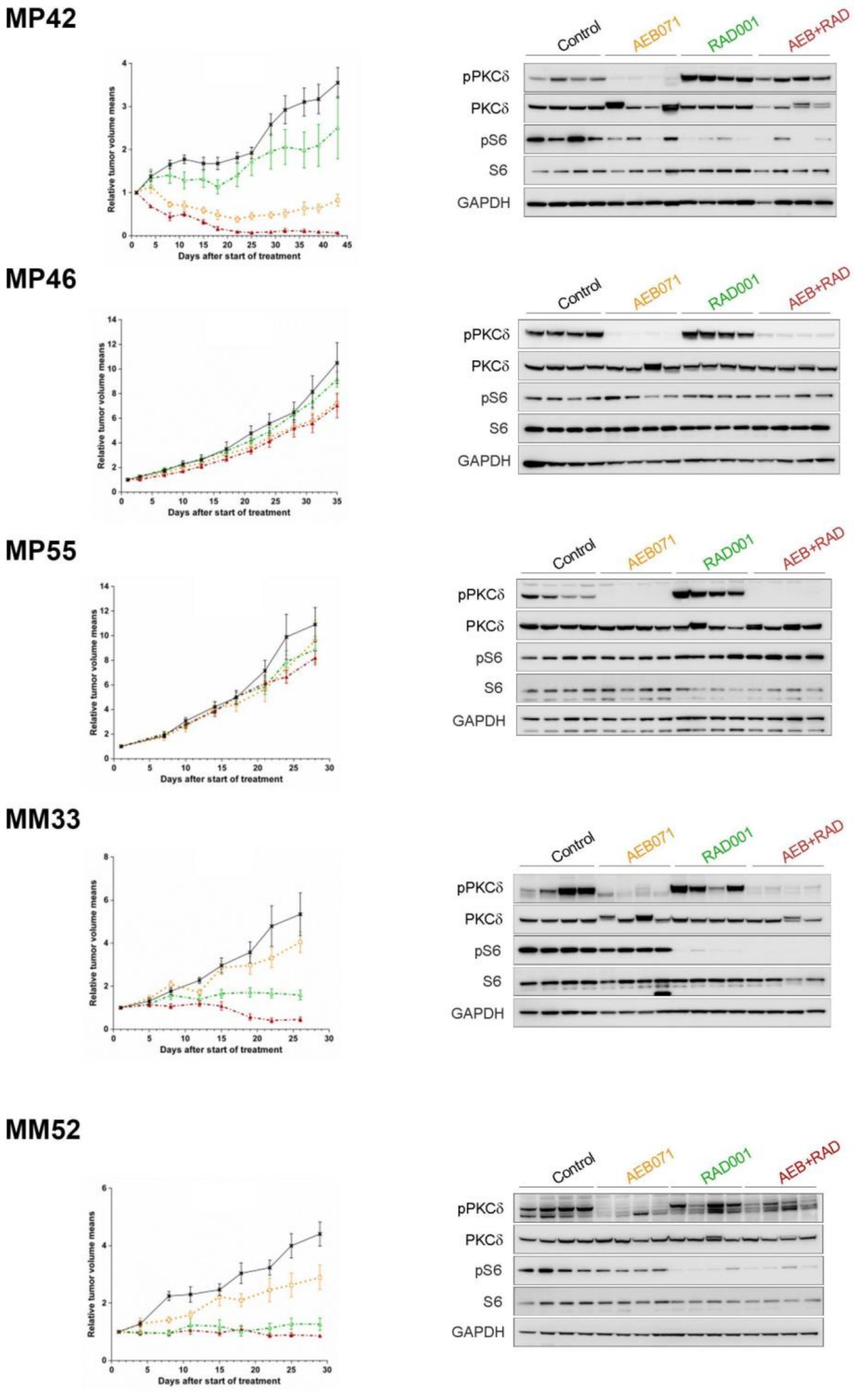

Figure S6: Pharmacodynamic marker analysis for the AEB071 + RAD001 combination. Samples were collected either 24h after the last dose (MP42, MM33, MM52) or 7h after the last injection of drugs (MP46, MP55). Four tumors per therapeutic group have been analyzed.

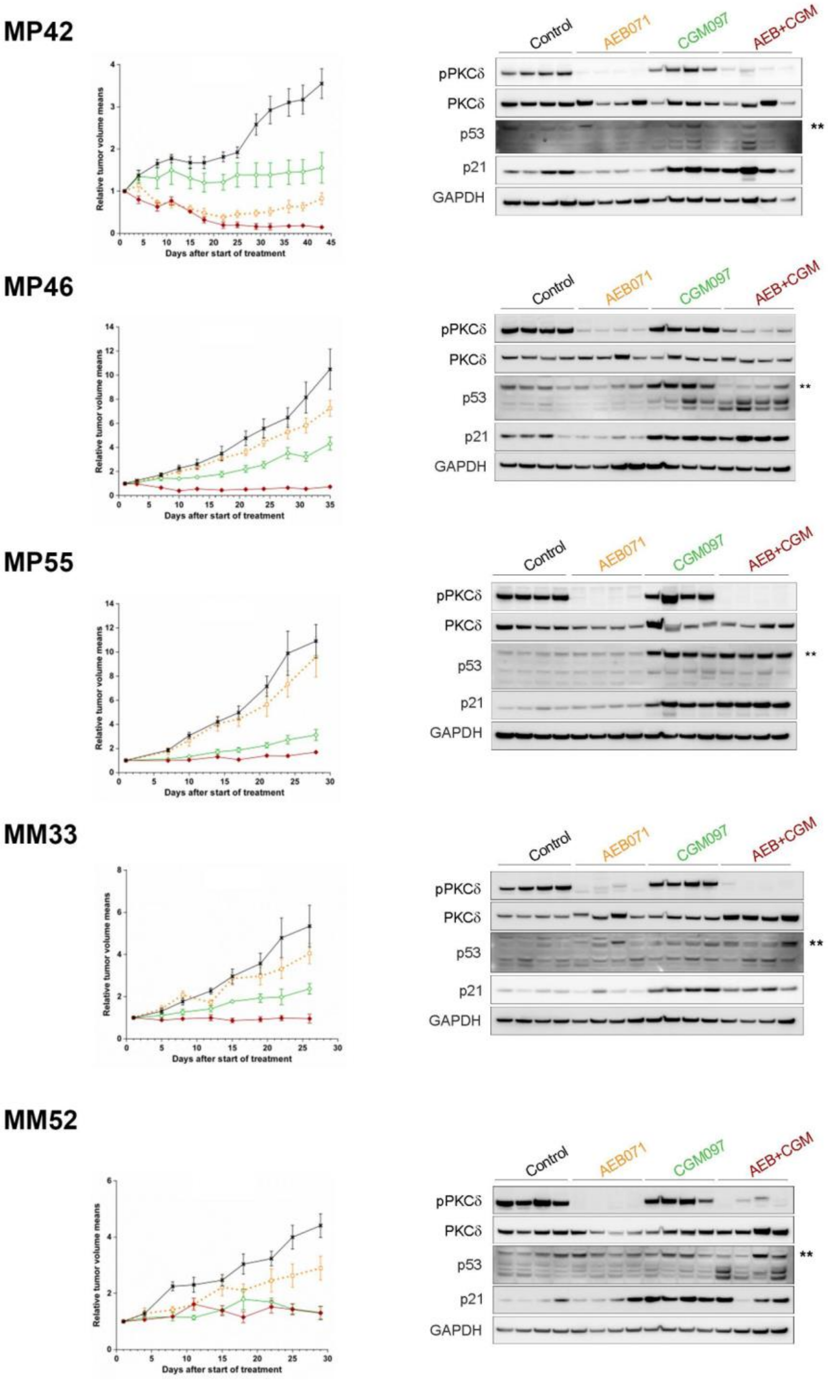

Figure S7: Pharmacodynamic marker analysis for the AEB071 + CGM097 combination. Samples were collected either 24h after the last dose (MP42, MM33, MM52) or 7h after the last injection of drugs (MP46, MP55). Four tumors per therapeutic group have been analyzed.

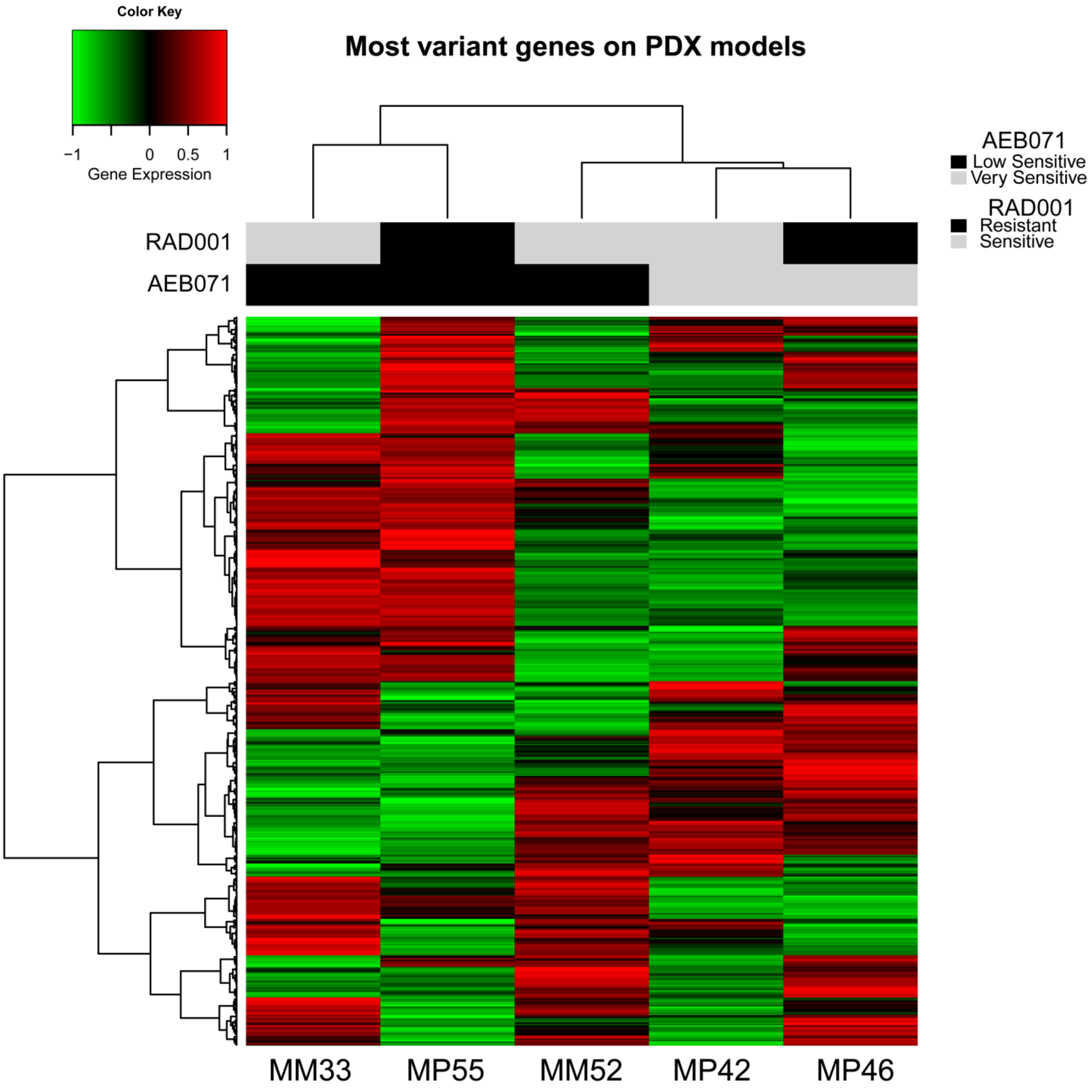

Figure S8: Heatmap of the most variant genes in UM PDXs. Hierarchical clustering (Ward method and Pearson correlation) based on 500 most variant genes (IQR) among the five PDX models at passage 1. Each sample was labelled according the response to AEB071 or RAD001. No correlation between clusters and pharmacological response to AEB071 or RAD001 was observed between sensitive and non-sensitive models.

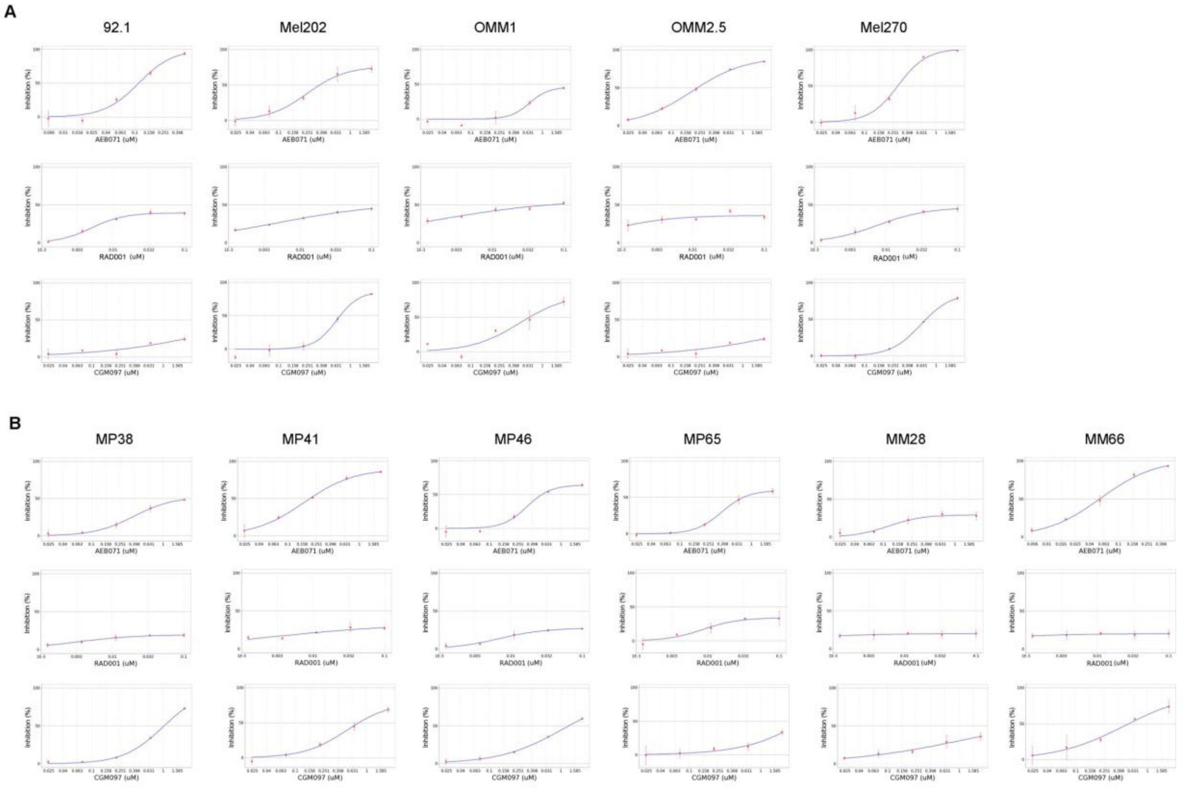

Figure S9: Dose response curves to AEB071, RAD001 and CGM097 in the *GNAQ/11* mutated UM cell lines used in this study. The curves correspond to response to single agent treatments done in Figures S12 and S13. Five 3-fold serial dilutions were tested. AEB071 was tested from 0 to 2 µM in all lines, except for 92.1 and MM66 cell lines for which the maximal dose was 0.5 µM. RAD001 and CGM097 were used from 0 to 0.1 µM and from 0 to 2 µM respectively. The averages between triplicate points were made and percentages of growth inhibition relative to DMSO-control treatment are represented ± SEM.

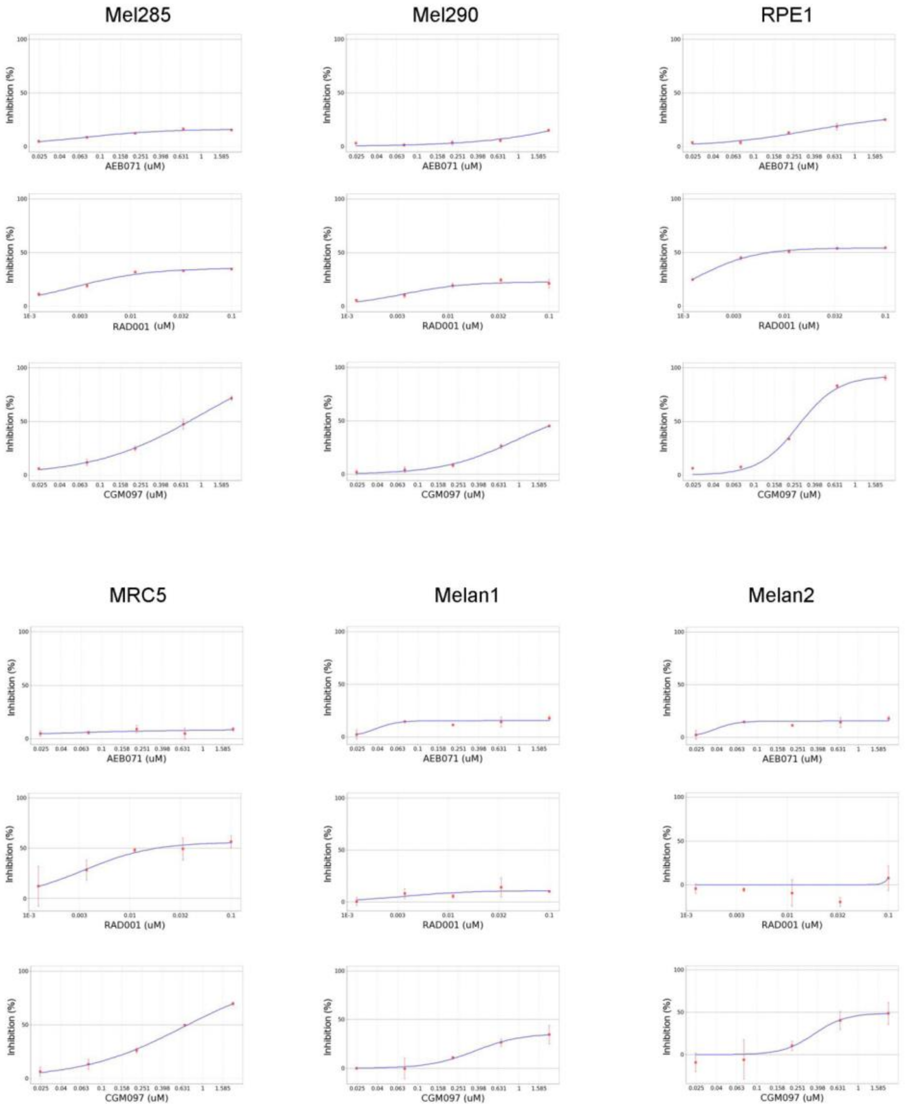

Figure S10: Dose response curves to AEB071, RAD001 and CGM097 in control cell lines. The curves correspond to response to single agent treatments done in Figures S12 and S13. Five 3-fold serial dilutions were tested. AEB071 was tested from 0 to 2 µM in all lines. RAD001 and CGM097 were used from 0 to 0.1 µM and from 0 to 2 µM respectively. The averages between triplicate points were made and percentages of growth inhibition relative to DMSO-control treatment are represented ± SEM.

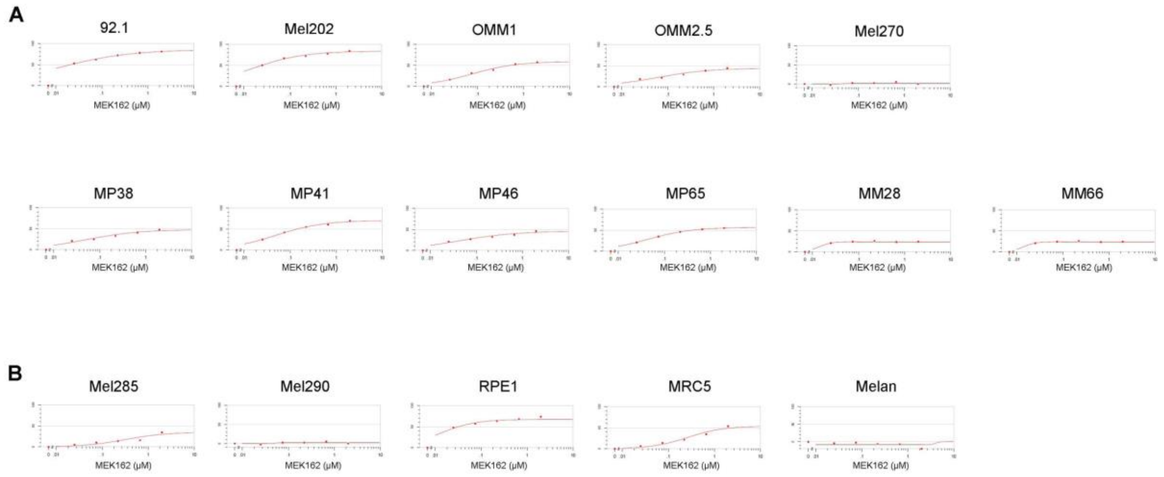

Figure S11: Dose response curves to MEK162 in the *GNAQ/11* mutated UM cell lines and control lines. The curves correspond to response to single agent treatments done in Figure S14. Five 3-fold serial dilutions were tested. AEB071 was tested from 0 to 2 µM in all lines, except for 92.1 and MM66 cell lines for which the maximal dose was 0.5 µM. MEK162 were used from 0 to 2 µM. The averages between triplicate points were made and percentages of growth inhibition relative to DMSO-control treatment are represented ± SEM.

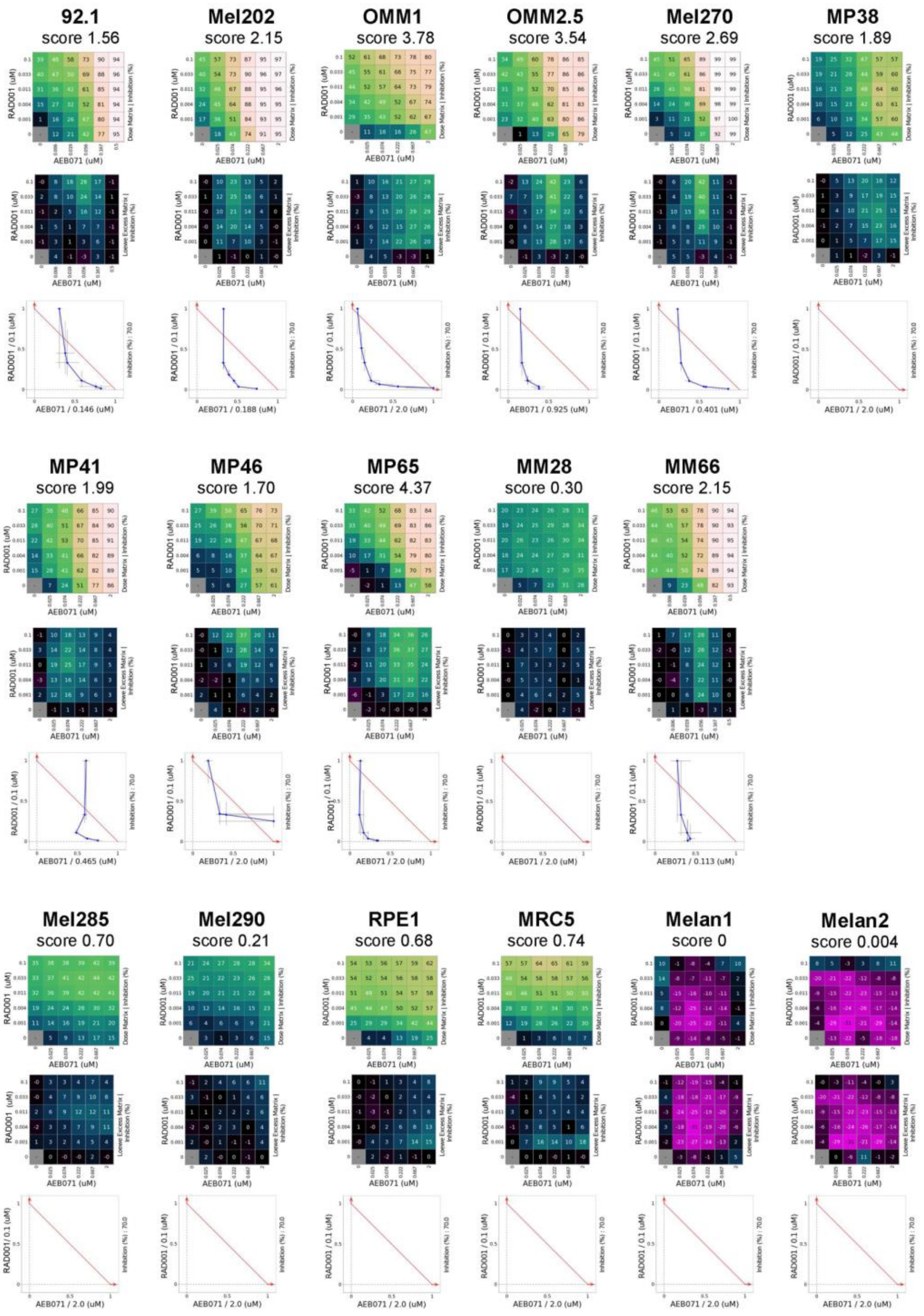

Figure S12: *In vitro* evaluation of AEB071 + RAD001 combination. For each cell line: *Top panel:* dose matrix with the percentage of growth inhibition compared to DMSO control. *Middle panel:* Loewe excess matrix with expected inhibition values according to the Loewe algorithm. *Bottom panel:* Isobolograms. The red line marks additivity, while experimental data are represented in blue.

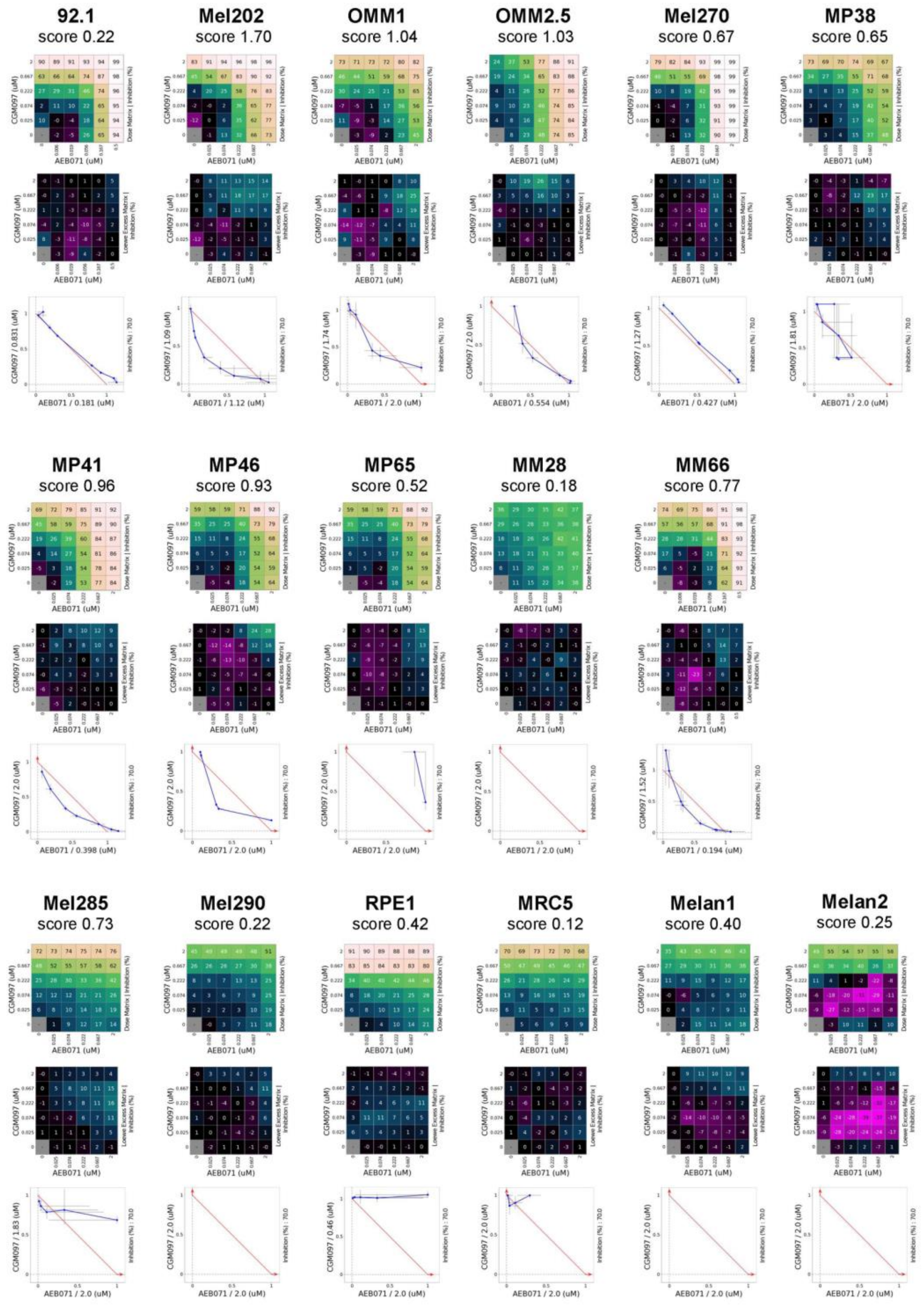

Figure S13: ***In vitro*** **evaluation of AEB071 + CGM097 combination.** For each cell line: *Top panel:* dose matrix with the percentage of growth inhibition compared to DMSO control. *Middle panel:* Loewe excess matrix with expected inhibition values according to the Loewe algorithm. *Bottom panel:* Isobolograms. The red line marks additivity, while experimental data are represented in blue.

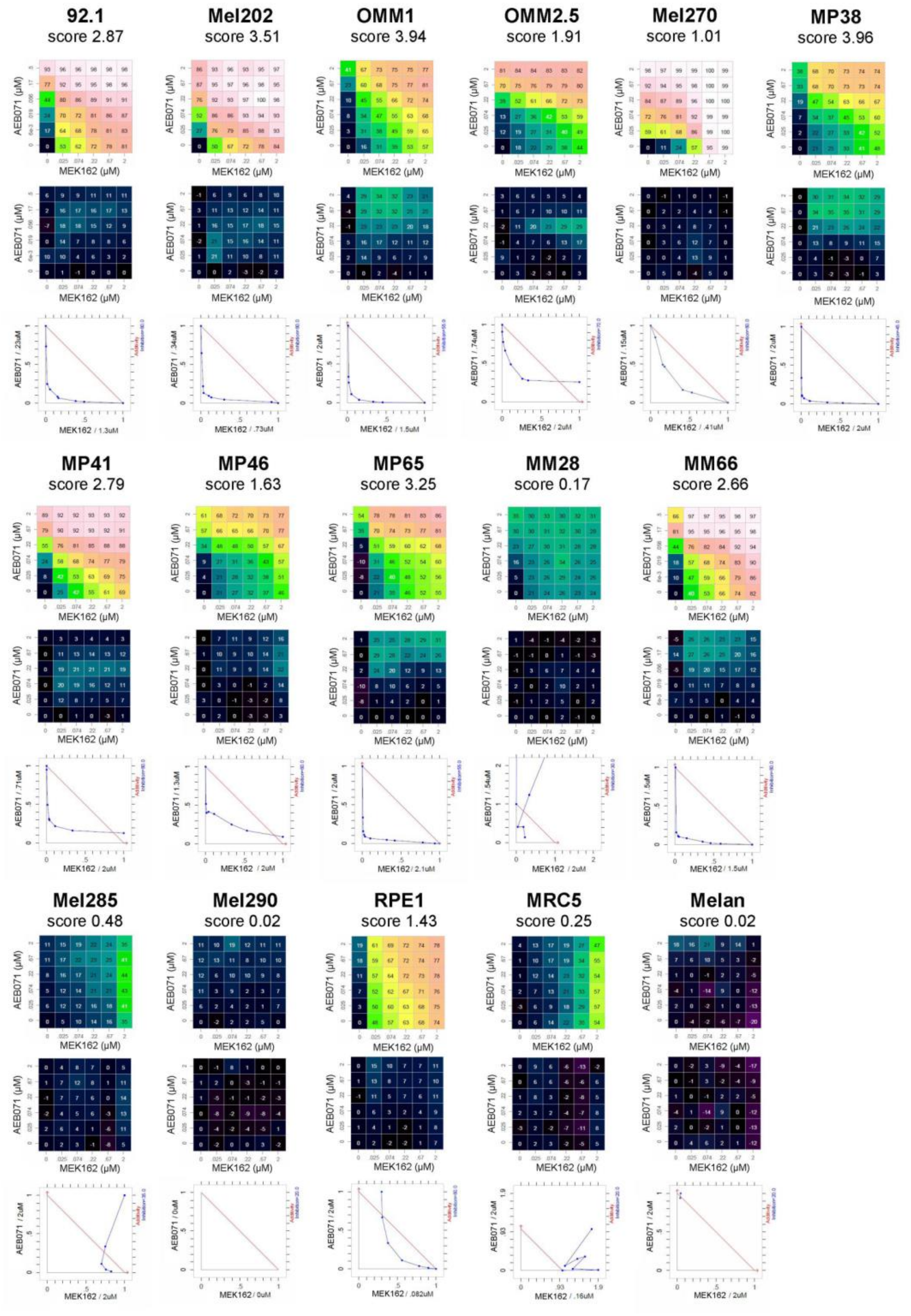

Figure S14: *In vitro* evaluation of AEB071 + MEK162 combination. For each cell line: *Top panel:* dose matrix with the percentage of growth inhibition compared to DMSO control. *Middle panel:* Loewe excess matrix with expected inhibition values according to the Loewe algorithm. *Bottom panel:* Isobolograms. The red line marks additivity, while experimental data are represented in blue.

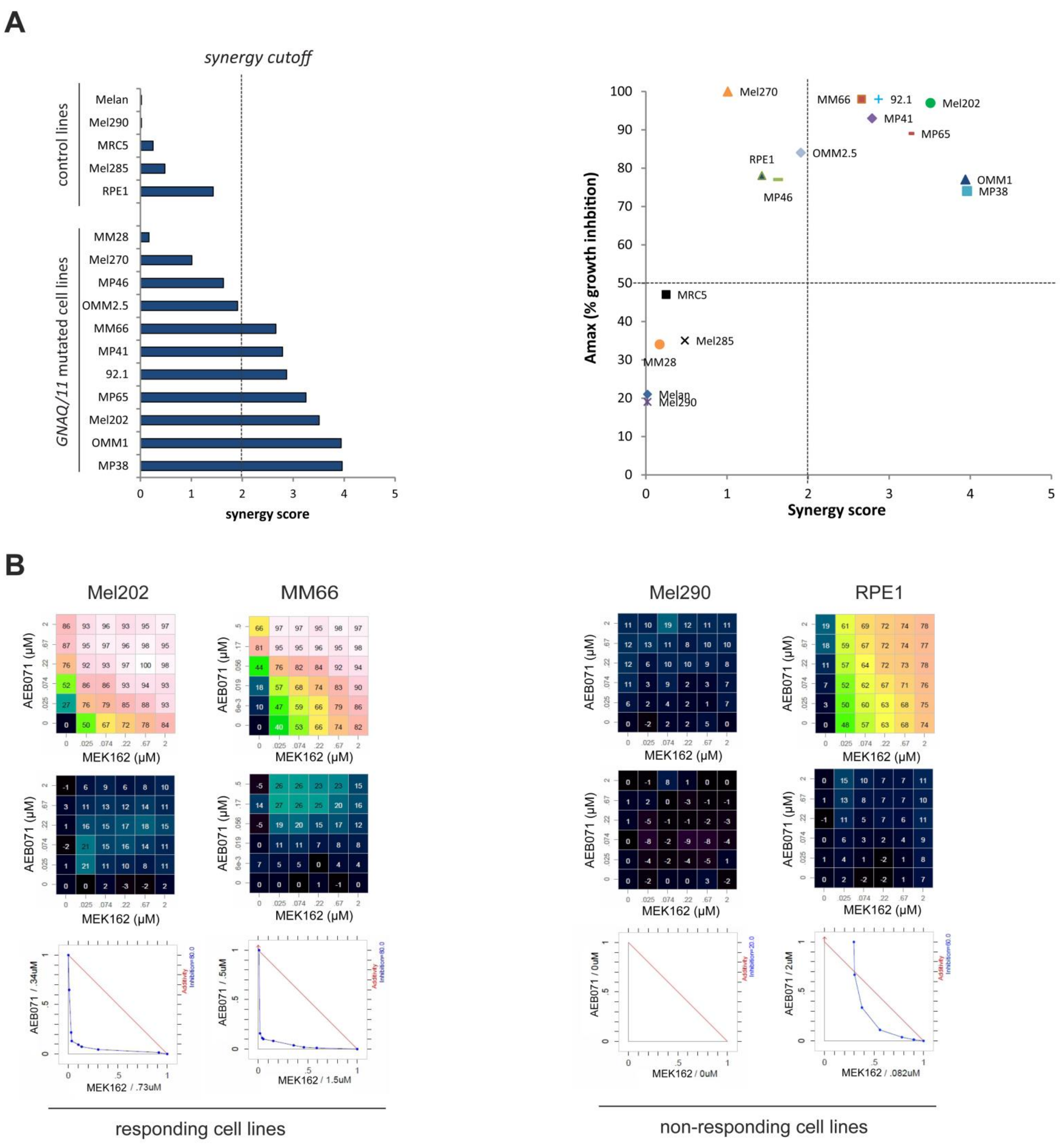

Figure S15: *In vitro* evaluation of AEB071 + MEK162 combination – continued. (A) *Left:* histogram ranking all tested cell lines according to their synergy score. *Right:* Dot Plot representing Amax values (y-axis) and synergy scores (x-axis) for all tested cell lines. **(B)** Examples of two cell lines with combination activity (Mel202 and MM66) and two cell lines with no combination activity (Mel290 and RPE1). The matrix representing percentage of growth inhibition *(top panels)*, the matrix with the Loewe Excess results *(middle panels)* and isobolograms *(bottom panels)* are shown. In the isobolograms, the expected additivity line is in red and experimental data are represented in blue.

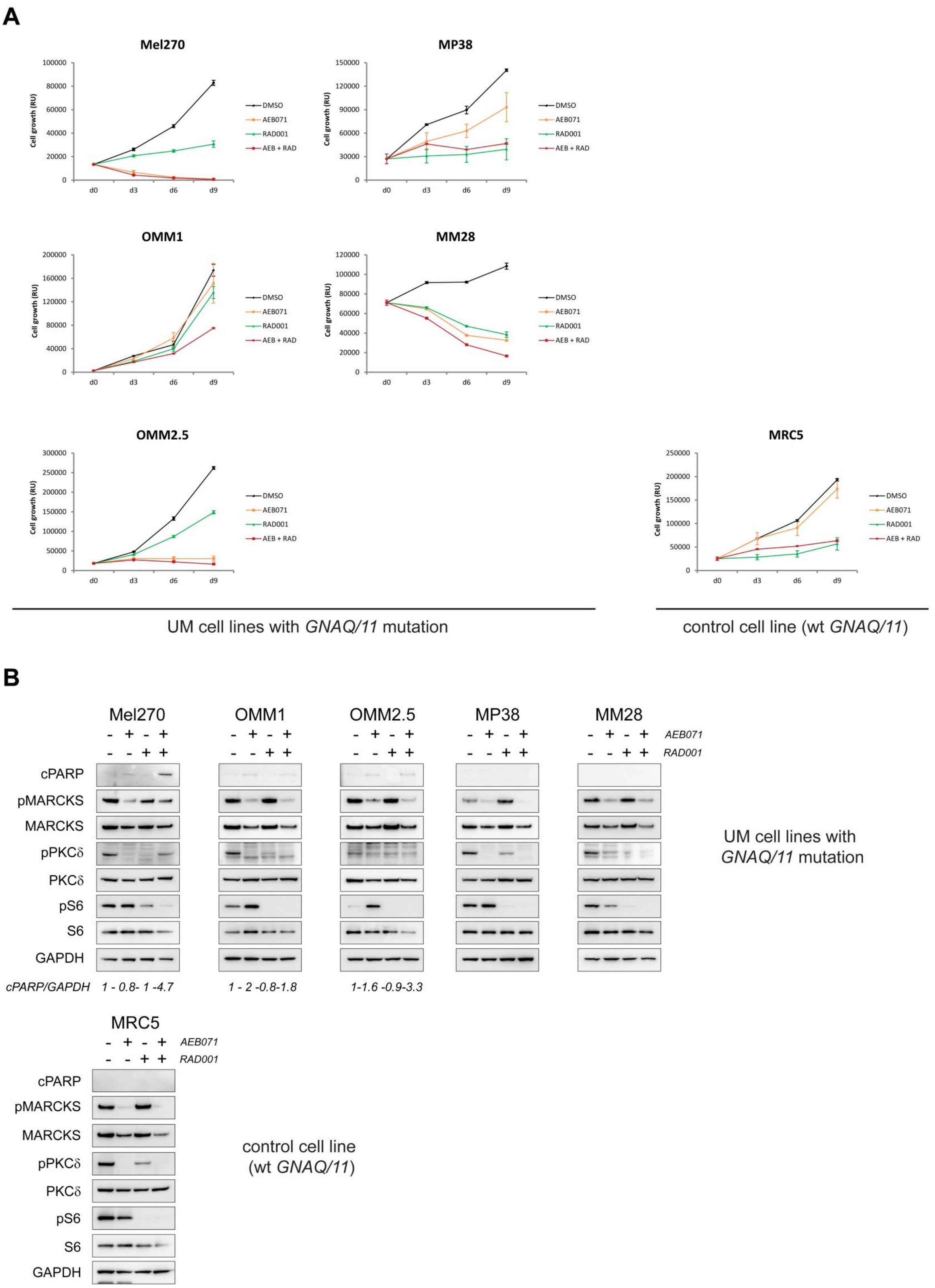

Figure S16: Co-inhibition of PKC and mTORC1 induces cell death in the majority of UM cell lines. This figure completes results shown in Figure 5. AEB071 (inhibition of PKC) and RAD001 (inhibition of mTORC1) were used respectively at 500 nM and 100 nM final

concentration. **(A)** Growth curve under treatment with AEB071 or/and RAD001. Cell viability was measured every 3 days with compound replacement at day 6. Averages between triplicates are represented ± SEM. **(B)** Molecular analyses by western blot. Apoptosis was assessed by cPARP. pMARCKS and pPKCd were used as pharmacodynamic markers for AEB071 activity, while pS6 was used as the marker for RAD001 activity.

Figure S17_*In vitro* evaluation of AEB071+CGM097 combination

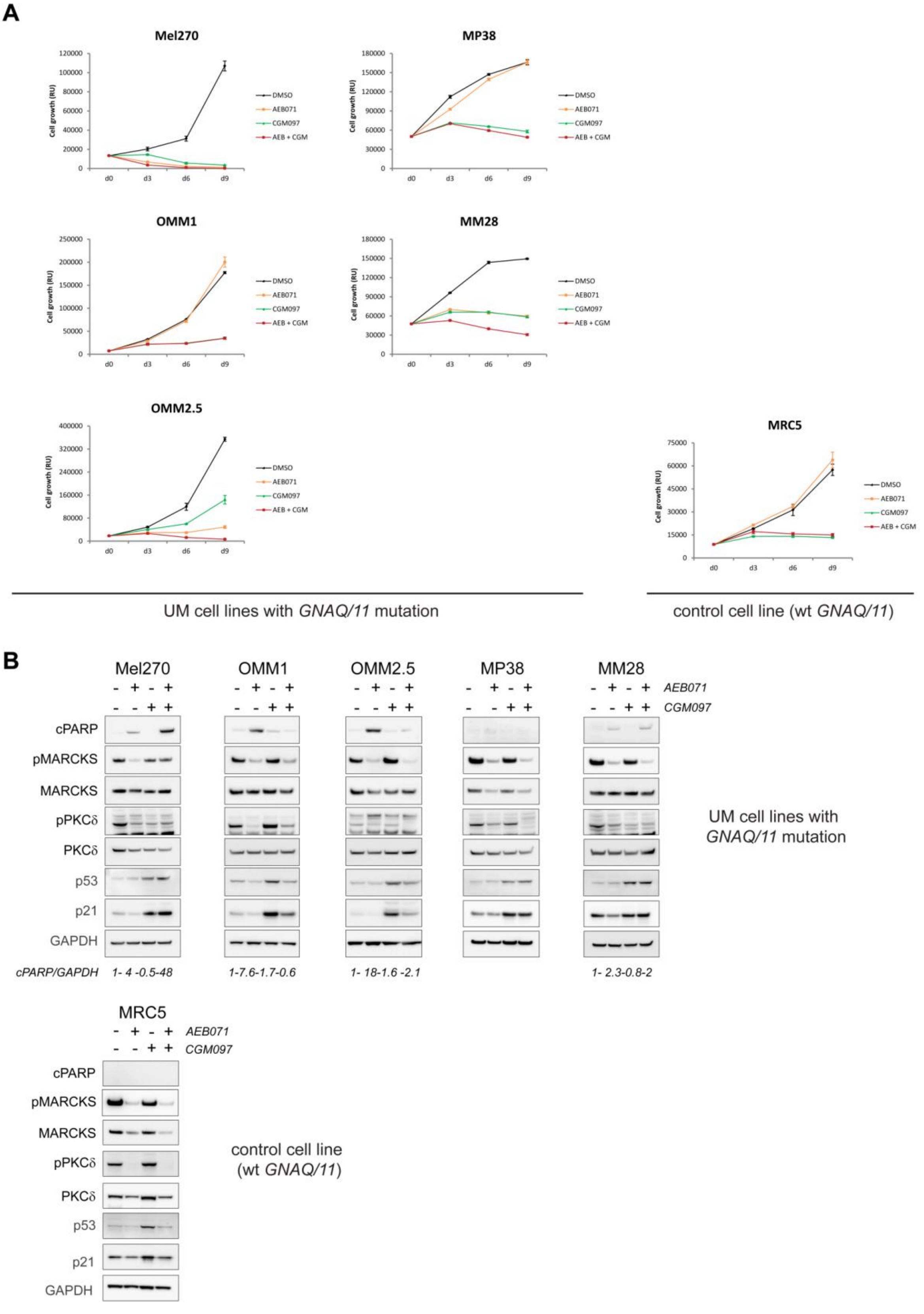

Figure S17: Co-inhibition of PKC and MDM2 induces cell death in the majority of UM cell lines. This figure completes results shown in Figure 6. AEB071 (inhibition of PKC) and CGM097 (inhibition of MDM2) were used respectively at 500 nM and 1 µM final concentration. **(A)** Growth curve under treatment with AEB071 or/and CGM097. Cell viability was measured every 3 days with compound replacement at day 6. Averages between triplicates are represented ± SEM. **(B)** Molecular analyses by western blot. Apoptosis was assessed by cPARP. pMARCKS and pPKCd were used as pharmacodynamic markers for AEB071 activity, while p53 and p21 were used as the marker for CGM097 activity.

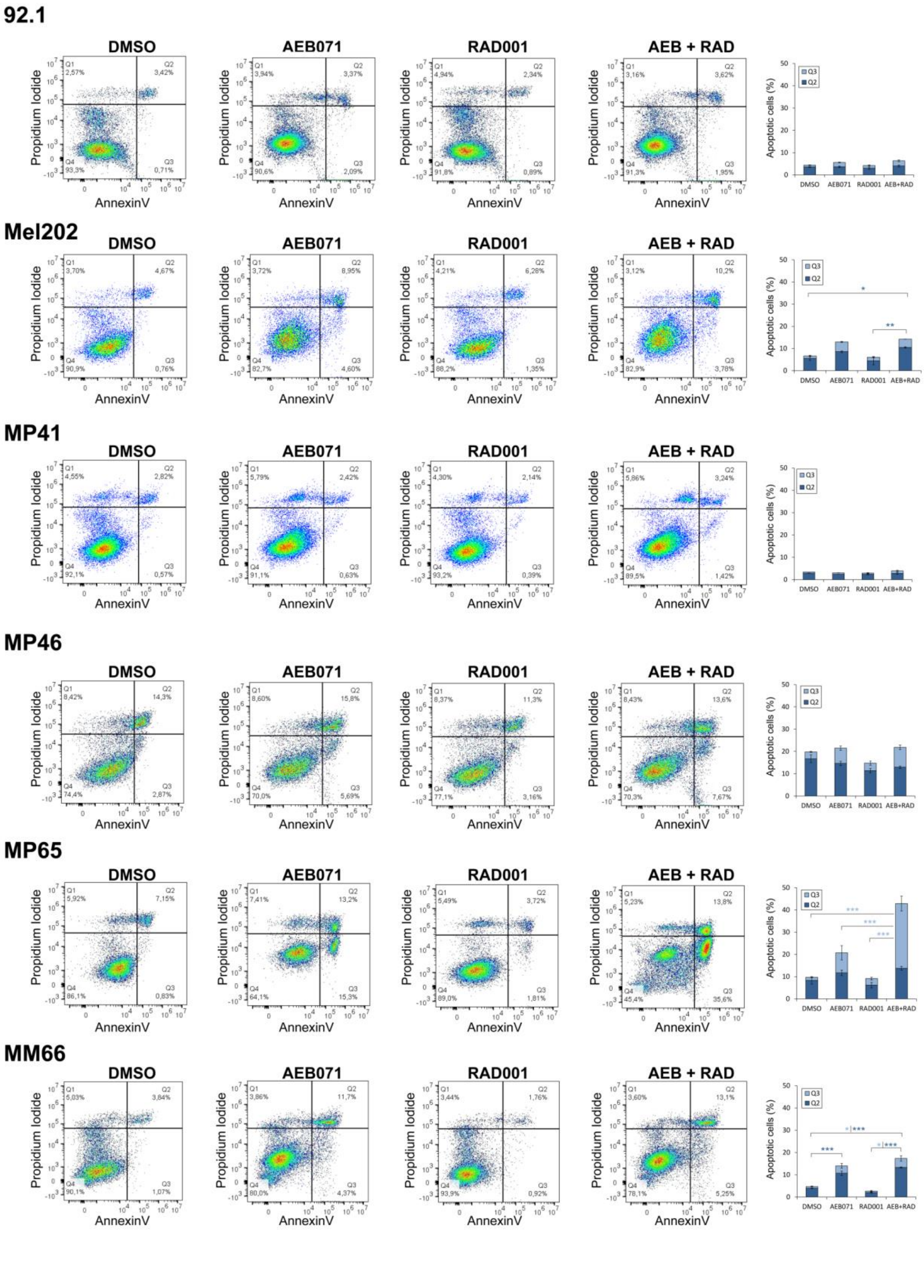
92.1
Mel202
MP41
MP46
MP65
MM66
DMSO
AEB071
RAD001
AEB + RAD
Propidium Iodide
AnnexinV
Q1
Q2
Q3
Q4
Apoptotic cells (%)
DMSO
AEB071
RAD001
AEB+RAD

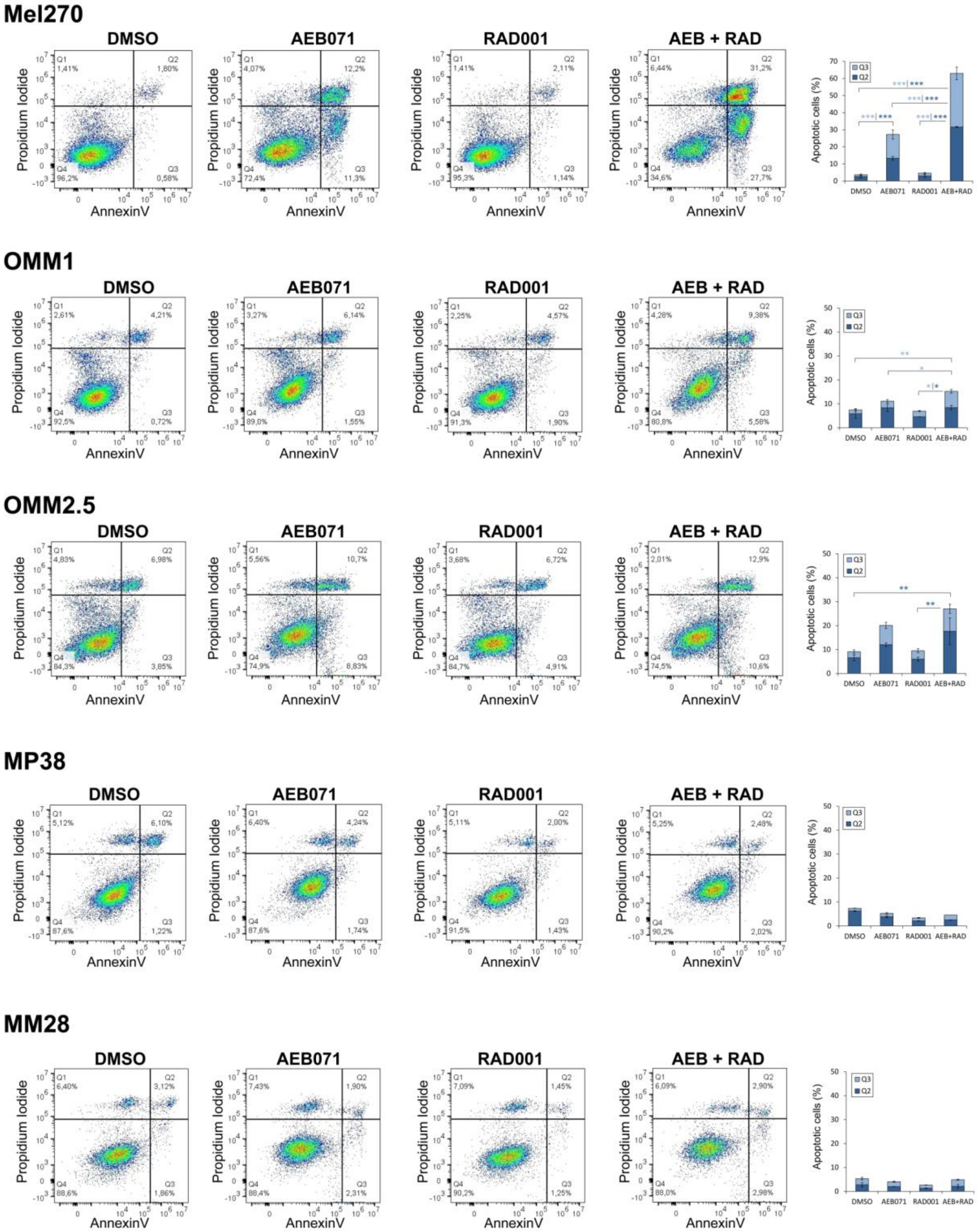
Mel270
DMSO
AEB071
RAD001
AEB + RAD
Propidium Iodide
AnnexinV
Q1 1,41% Q2 1,80% Q4 96,2% Q3 0,58%
Q1 4,07% Q2 12,2% Q4 72,4% Q3 11,3%
Q1 1,41% Q2 2,11% Q4 95,3% Q3 1,14%
Q1 6,44% Q2 31,2% Q4 34,6% Q3 27,7%
Apoptotic cells (%)
Q3
Q2
DMSO AEB071 RAD001 AEB+RAD
OMM1
Q1 2,61% Q2 4,21% Q4 92,5% Q3 0,72%
Q1 3,27% Q2 6,14% Q4 89,0% Q3 1,55%
Q1 2,25% Q2 4,57% Q4 91,3% Q3 1,90%
Q1 4,28% Q2 9,38% Q4 80,8% Q3 5,58%
OMM2.5
Q1 4,83% Q2 6,98% Q4 84,3% Q3 3,85%
Q1 5,56% Q2 10,7% Q4 74,9% Q3 8,83%
Q1 3,68% Q2 6,72% Q4 84,7% Q3 4,91%
Q1 2,01% Q2 12,9% Q4 74,5% Q3 10,6%
MP38
Q1 5,12% Q2 6,10% Q4 87,6% Q3 1,22%
Q1 6,40% Q2 4,24% Q4 87,6% Q3 1,74%
Q1 5,11% Q2 2,00% Q4 91,5% Q3 1,43%
Q1 5,25% Q2 2,48% Q4 90,2% Q3 2,02%
MM28
Q1 6,40% Q2 3,12% Q4 88,6% Q3 1,86%
Q1 7,43% Q2 1,90% Q4 88,4% Q3 2,31%
Q1 7,09% Q2 1,45% Q4 90,2% Q3 1,25%
Q1 6,09% Q2 2,90% Q4 88,0% Q3 2,98%

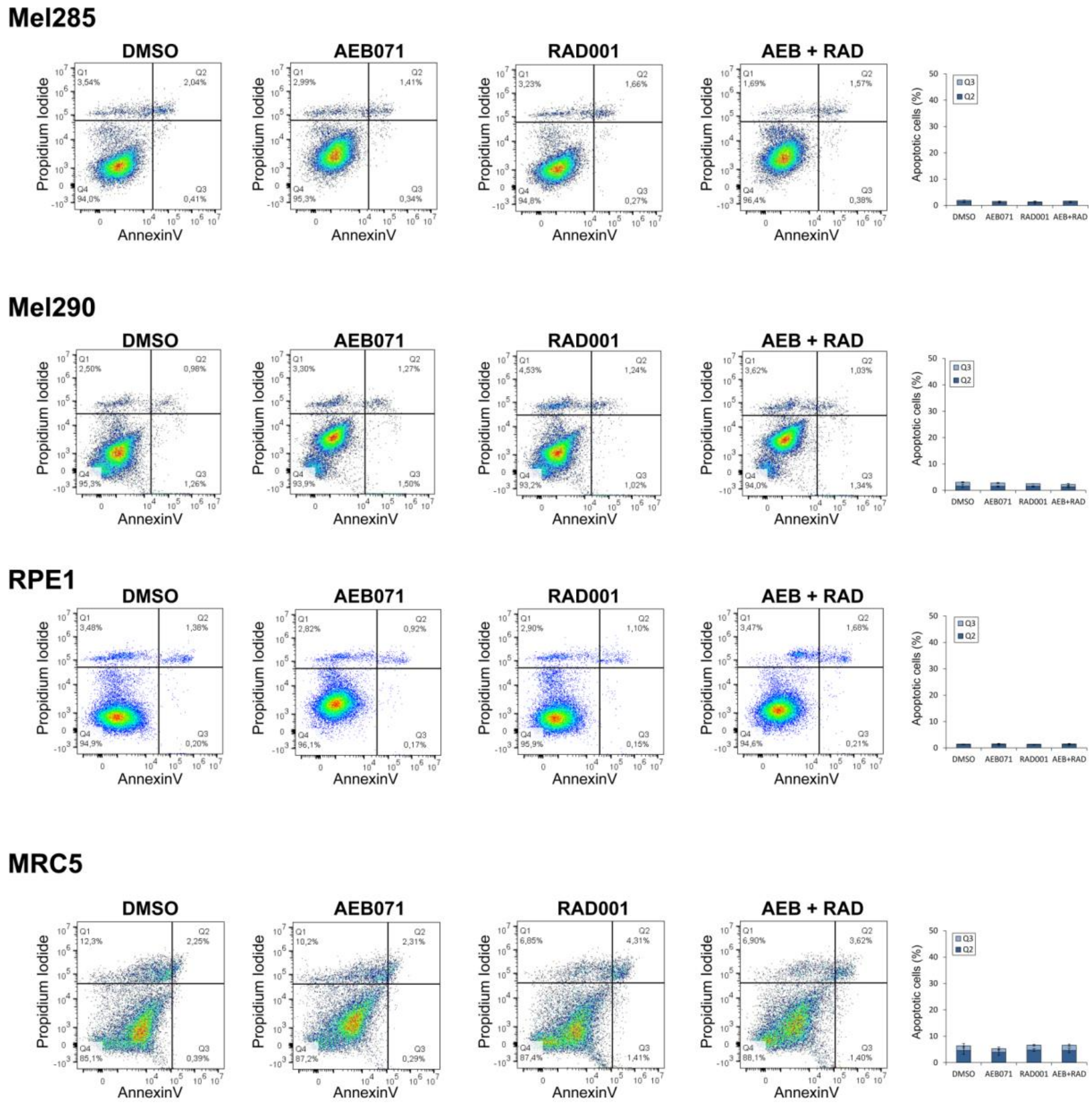

Figures S18-19-20: Quantification of apoptosis by Annexin V after PKC and mTORC1 inhibition alone or in combination. Results of one representative experiment are shown. Results of flow cytometry analyses are represented with the percentages of cells in each population. Q1: necrotic cells. Q2: late apoptotic cells. Q3: early apoptotic cells. Q4: alive cells. Quantification of all apoptotic cells (Q2 and Q3) was done and represented in the graph on the left of each cell line panel. Results of two to three independent experiments are combined and shown as mean ±SEM. *p<0.05, **p<0.01, ***p<0.001 by two-way ANOVA with Bonferroni correction. The color of the * matches the color chosen for either Q2 or Q3.

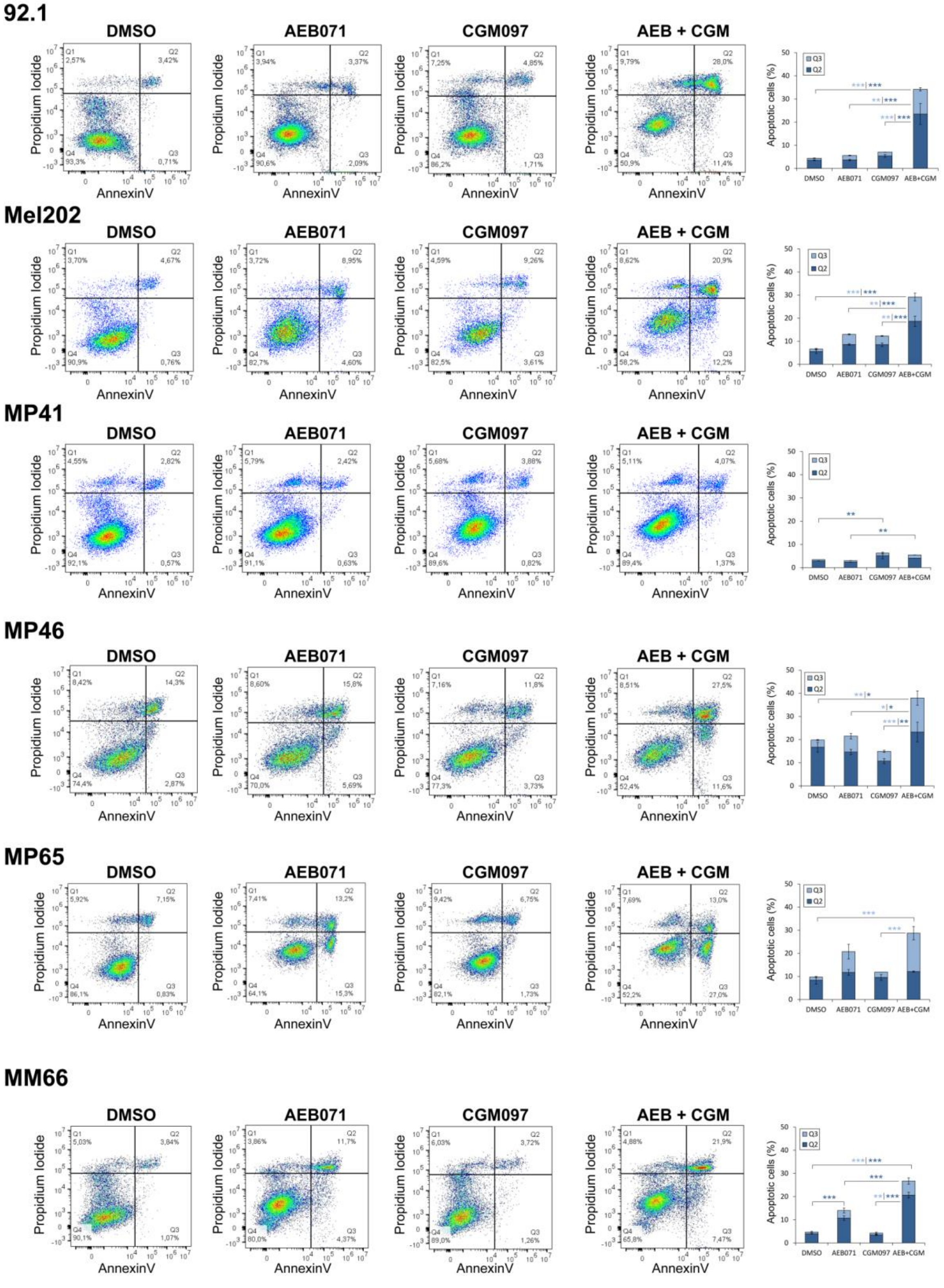
92.1
Mel202
MP41
MP46
MP65
MM66
DMSO
AEB071
CGM097
AEB + CGM
Propidium Iodide
AnnexinV
Q1
Q2
Q3
Q4
Apoptotic cells (%)
DMSO
AEB071
CGM097
AEB+CGM

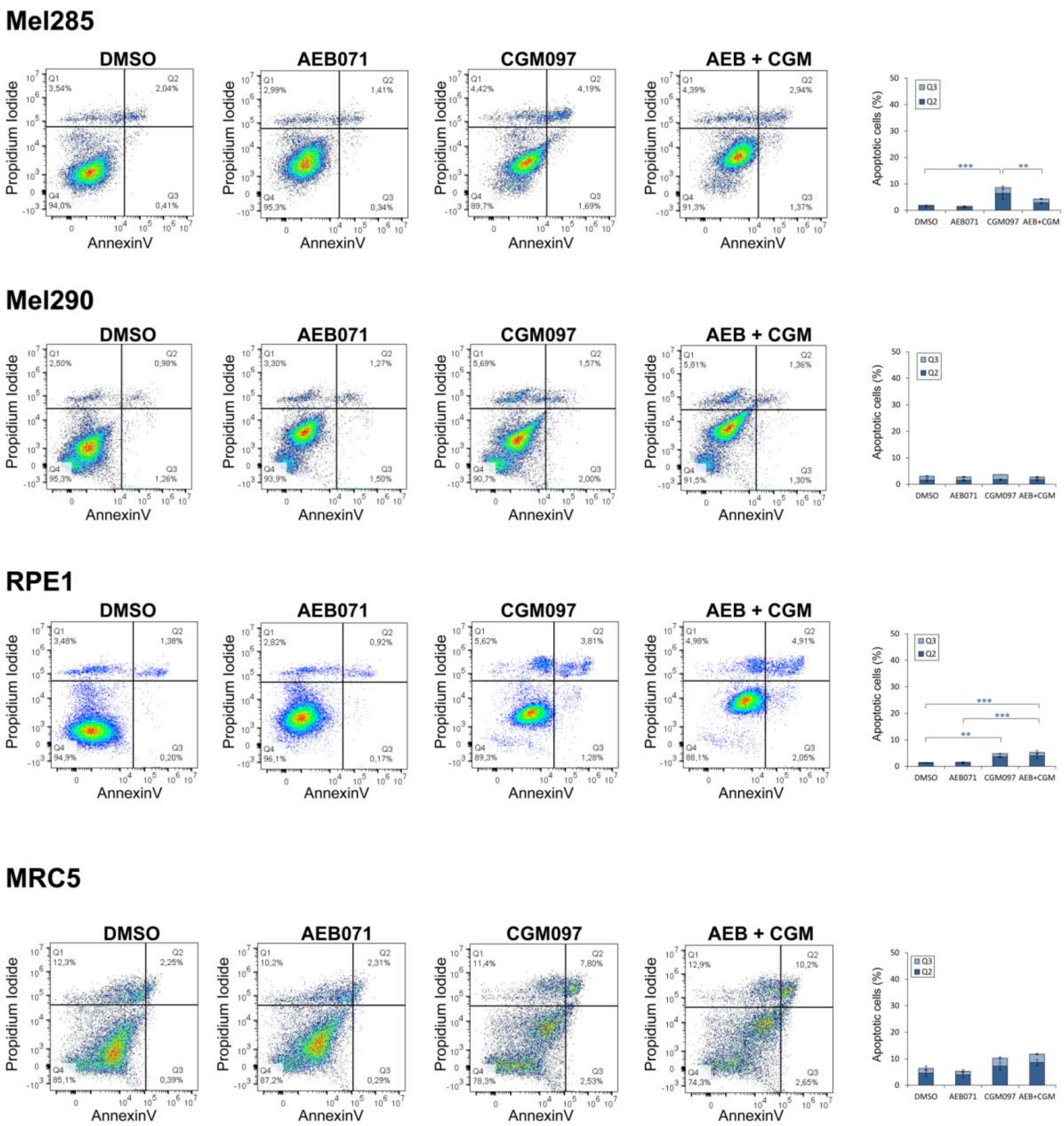
Mel285
DMSO
AEB071
CGM097
AEB + CGM
Q1 3,54%
Q2 2,04%
Q4 94,0%
Q3 0,41%
Q1 2,99%
Q2 1,41%
Q4 95,3%
Q3 0,34%
Q1 4,42%
Q2 4,19%
Q4 89,7%
Q3 1,69%
Q1 4,39%
Q2 2,94%
Q4 91,3%
Q3 1,37%
Propidium Iodide
AnnexinV
Apoptotic cells (%)
Q3
Q2
DMSO
AEB071
CGM097
AEB+CGM
***
**
Mel290
Q1 2,50%
Q2 0,98%
Q4 95,3%
Q3 1,26%
Q1 3,30%
Q2 1,27%
Q4 93,9%
Q3 1,50%
Q1 5,69%
Q2 1,57%
Q4 90,7%
Q3 2,00%
Q1 5,81%
Q2 1,36%
Q4 91,5%
Q3 1,30%
RPE1
Q1 3,48%
Q2 1,38%
Q4 94,9%
Q3 0,20%
Q1 2,82%
Q2 0,92%
Q4 96,1%
Q3 0,17%
Q1 5,62%
Q2 3,81%
Q4 89,3%
Q3 1,28%
Q1 4,98%
Q2 4,91%
Q4 88,1%
Q3 2,05%
MRC5
Q1 12,3%
Q2 2,25%
Q4 85,1%
Q3 0,39%
Q1 10,2%
Q2 2,31%
Q4 87,2%
Q3 0,29%
Q1 11,4%
Q2 7,80%
Q4 78,3%
Q3 2,53%
Q1 12,9%
Q2 10,2%
Q4 74,3%
Q3 2,65%

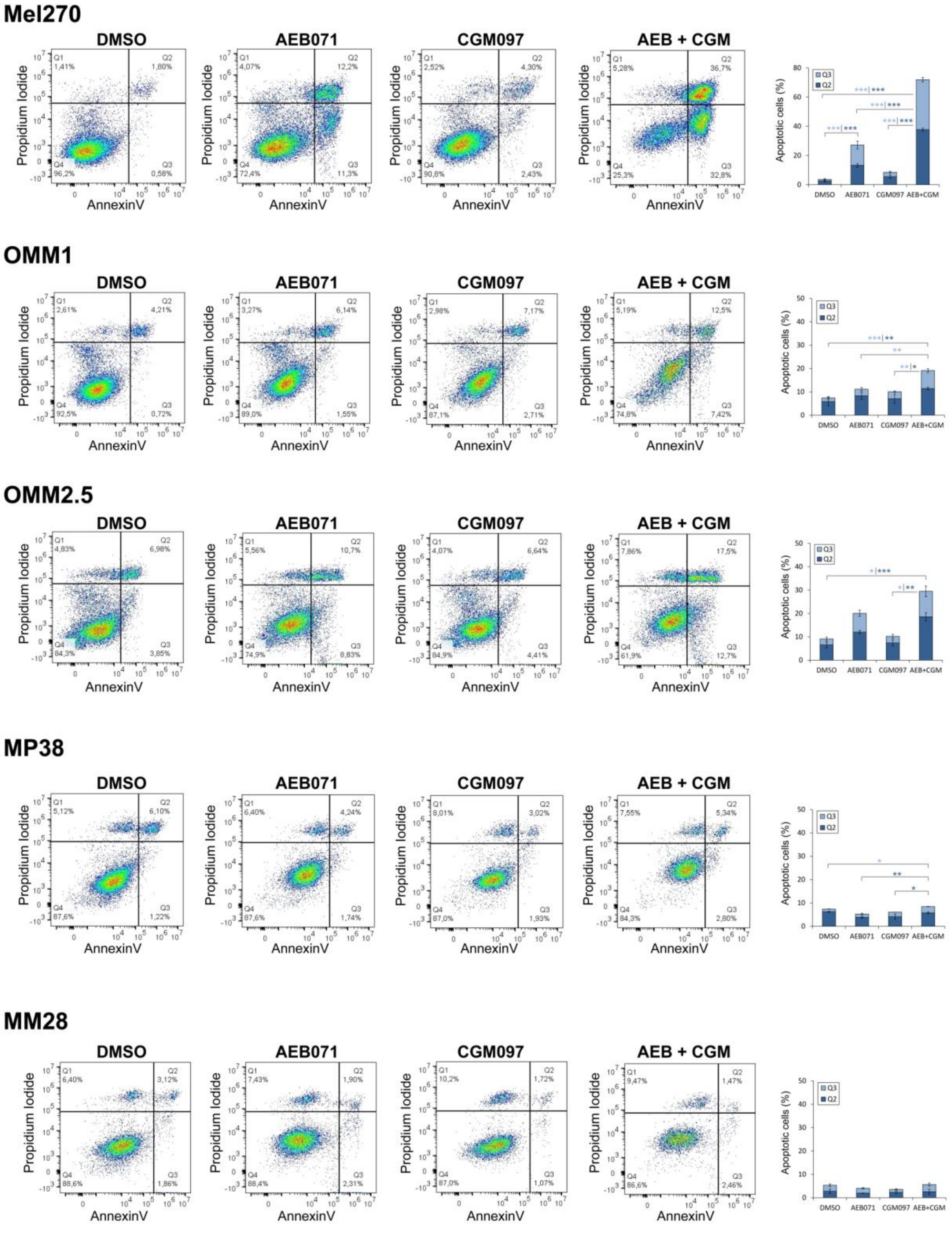

Figures S21-22-23: Quantification of apoptosis by Annexin V after PKC and p53-MDM2 inhibition alone or in combination. Results of one representative experiment are shown. Results of flow cytometry analyses are represented with the percentages of cells in each population. Q1: necrotic cells. Q2: late apoptotic cells. Q3: early apoptotic cells. Q4: alive

cells. Quantification of all apoptotic cells (Q2 and Q3) was done and represented in the graph on the left of each cell line panel. Results of two to three independent experiments are combined and shown as mean ±SEM. *p<0.05, **p<0.01, ***p<0.001 by two-way ANOVA with Bonferroni correction. The color of the * matches the color chosen for either Q2 or Q3.
